# In cellulo crystallization of *Trypanosoma brucei* IMP dehydrogenase enables the identification of genuine co-factors

**DOI:** 10.1038/s41467-020-14484-w

**Published:** 2020-01-30

**Authors:** Karol Nass, Lars Redecke, M. Perbandt, O. Yefanov, M. Klinge, R. Koopmann, F. Stellato, A. Gabdulkhakov, R. Schönherr, D. Rehders, J. M. Lahey-Rudolph, A. Aquila, A. Barty, S. Basu, R. B. Doak, R. Duden, M. Frank, R. Fromme, S. Kassemeyer, G. Katona, R. Kirian, H. Liu, I. Majoul, J. M. Martin-Garcia, M. Messerschmidt, R. L. Shoeman, U. Weierstall, S. Westenhoff, T. A. White, G. J. Williams, C. H. Yoon, N. Zatsepin, P. Fromme, M. Duszenko, H. N. Chapman, C. Betzel

**Affiliations:** 1 Center for Free-Electron Laser Science (CFEL), Deutsches Elektronen-Synchrotron DESY, Notkestr. 85, 22607 Hamburg, Germany; 2 Joint Laboratory for Structural Biology of Infection and Inflammation, Institute of Biochemistry and Molecular Biology, University of Hamburg, and Institute of Biochemistry, University of Lübeck, at Deutsches Elektronen-Synchrotron (DESY), Notkestr. 85, 22607 Hamburg, Germany; 3 German Centre for Infection Research, University of Lübeck, 23562 Lübeck, Germany; 4 Institute of Biochemistry and Molecular Biology, University of Hamburg, at Deutsches Elektronen-Synchrotron (DESY), Notkestr. 85, 22607 Hamburg, Germany; 5 The Hamburg Centre for Ultrafast Imaging (CUI), Luruper Chaussee 149, 22761 Hamburg, Germany; 6 Interfaculty Institute of Biochemistry, University of Tübingen, Hoppe-Seyler-Str.4, 72076 Tübingen, Germany; 7 Institute of Protein Research, Russian Academy of Sciences, 4 Institutskaya Str., Pushchino, Moscow Region Russia 142290; 8 Institute of Biochemistry, University of Lübeck, Ratzeburger Allee 160, 23562 Lübeck, Germany; 9 Deutsches Elektronen Synchrotron (DESY), Photon Science, Notkestr. 85, 22607 Hamburg, Germany; 10 Department of Chemistry and Biochemistry, Arizona State University, Tempe, AZ 85287-160 USA; 11 Department of Physics, Arizona State University, Tempe, AZ 85411 USA; 12 Institute of Biology, University of Lübeck, Ratzeburger Allee 160, 23562 Lübeck, Germany; 13 Biology and Biotechnology Division, Physical & Life Sciences Directorate, Lawrence Livermore National Laboratory, 7000 East Avenue, Livermore, CA 94550 USA; 14 Max-Planck-Institute for Medical Research, Jahnstr. 29, 69120 Heidelberg, Germany; 15 Department of Chemistry and Molecular Biology, University of Gothenburg, 40530 Gothenburg, Sweden; 16 Center for Applied Structural Discovery (CASD), Biodesign Institute, Arizona State University, 727 East Tyler Street, Tempe, AZ 85287 USA; 17 LCLS, SLAC National Accelerator Laboratory, 2575 Sand Hill Road, Menlo Park, CA 94025 USA; 18 Institute of Neurophysiology, University of Tübingen, Keplerstr. 15, 72074 Tübingen, Germany; 19 Department of Physics, University of Hamburg, Luruper Chaussee 149, 22761 Hamburg, Germany; 20 Present Address: Paul Scherrer Institute (PSI), Forschungstrasse 111, 5232 Villigen, PSI Switzerland; 21 Present Address: Institute of Biochemistry, University of Lübeck, Ratzeburger Allee 160, 23562 Lübeck, Germany; 22 Present Address: Deutsches Elektronen Synchrotron (DESY), Photon Science, Notkestr. 85, 22607 Hamburg, Germany; 23 Present Address: BioAgilytix Europe GmbH, Lademannbogen 10, 22339 Hamburg, Germany; 24 Present Address: Dipartimento di Fisica, Università di Roma Tor Vergata and INFN, Via della Ricerca Scientifica 1, 00133 Rome, Italy; 25 Present Address: BODE Chemie GmbH, Melanchthonstraße 27, 22525 Hamburg, Germany; 26 Present Address: LCLS, SLAC National Accelerator Laboratory, 2575 Sand Hill Road, Menlo Park, CA 94025 USA; 27 Present Address: European Molecular Biology Laboratory (EMBL), Grenoble Outstation, 71 Avenue des Martyrs, CS 90181, 38042 Grenoble Cedex 9, Grenoble, France; 28 Present Address: Max Planck Institute for Medical Research, Jahnstr. 29, 69120 Heidelberg, Germany; 29 Present Address: Complex Systems Division, Beijing Computational Science Research Center, 100193 Beijing, China; 30 Present Address: Center for Applied Structural Discovery (CASD), Biodesign Institute, Arizona State University, 727 East Tyler Street, Tempe, AZ 85287 USA; 31 Present Address: Brookhaven National Laboratory (BNL), PO Box 5000, Upton, NY 11973-5000 USA; 32 Present Address: LCLS, SLAC National Accelerator Laboratory, 2575 Sand Hill Road, Menlo Park, CA 94025 USA; 33 Present Address: ARC Centre of Excellence in Advanced Molecular Imaging, Department of Chemistry and Physics, La Trobe Institute for Molecular Science, La Trobe University, Victoria, 3086 Australia

**Keywords:** Biochemistry, X-ray crystallography

## Abstract

Sleeping sickness is a fatal disease caused by the protozoan parasite *Trypanosoma brucei* (Tb). Inosine-5’-monophosphate dehydrogenase (IMPDH) has been proposed as a potential drug target, since it maintains the balance between guanylate deoxynucleotide and ribonucleotide levels that is pivotal for the parasite. Here we report the structure of TbIMPDH at room temperature utilizing free-electron laser radiation on crystals grown in living insect cells. The 2.80 Å resolution structure reveals the presence of ATP and GMP at the canonical sites of the Bateman domains, the latter in a so far unknown coordination mode. Consistent with previously reported IMPDH complexes harboring guanosine nucleotides at the second canonical site, TbIMPDH forms a compact oligomer structure, supporting a nucleotide-controlled conformational switch that allosterically modulates the catalytic activity. The oligomeric TbIMPDH structure we present here reveals the potential of in cellulo crystallization to identify genuine allosteric co-factors from a natural reservoir of specific compounds.

## Abstract (web summary)

*Trypanosoma brucei* inosine-5′-monophosphate dehydrogenase (IMPDH) is an enzyme in the guanine nucleotide biosynthesis pathway and of interest as a drug target. Here the authors present the 2.8 Å room temperature structure of TbIMPDH determined by utilizing X-ray free-electron laser radiation and crystals that were grown in insect cells and find that ATP and GMP are bound at the canonical sites of the Bateman domains.

## Introduction

Recent developments in serial crystallography data collection strategies at both X-ray free-electron lasers (XFELs) and synchrotron sources have paved the way for the use of protein crystals with dimensions in the nano- to low-micrometer size-range as suitable targets for X-ray crystallography^1–3^. XFELs produce radiation bursts of previously inaccessible brilliance and femtosecond duration that allow outrunning most radiation damage processes^4–6^. As a consequence of the improved X-ray intensities, also small crystals formed within living cells, denoted as ‘in cellulo crystals’ or ‘in vivo grown crystals’, became interesting for structural biologists. As known for more than a century, intracellular protein crystallization represents a native process that can provide distinct advantageous functions for the organism, mainly associated with storage, protection, and solid state catalysis, while abnormal crystalline states of usually soluble proteins have been identified as a pathogenic hallmark (reviewed in Schönherr et al.^7^). However, the crowded environment in the living cell was supposed to prevent the growth of sufficiently ordered crystals^8^, but particularly the small size prevented the use of in cellulo crystals as targets for structural biology over decades. In 2007, the first structure of a natively crystallizing protein, cypoviral polyhedrin, was solved using synchrotron radiation^9^, followed by several other successful examples of natively in cellulo crystallizing proteins up to now^7^.

Increasing evidence highlights that recombinant proteins can also form intracellular crystals within host cells during heterogeneous gene expression, a phenomenon predominantly observed in animal and baculovirus-infected insect cells^7,10^. The first high-resolution structure determined from in cellulo grown crystals formed by a recombinant, non-naturally crystallizing protein, was cathepsin B, a protease from the sleeping sickness-causing parasite *Trypanosoma brucei* (Tb)^11^. Depending on the size of the crystals, both synchrotron and XFEL radiation has nowadays been used several-fold to elucidate structural information from recombinant proteins that form intracellular microcrystals, e.g., the coral fluorescent protein Xpa^12^, the metazoan-specific kinase PAK4^13^, and the BinAB larvicide from *Lysinibacillus sphaericus*^14^. However, it is still not clear if the phenomenon of in cellulo crystallization is restricted to a limited number of proteins that are evolutionary optimized for native crystal formation, or if living cells can be systematically exploited as crystal factories for a large number of recombinant proteins, when the associated cellular processes have been fully understood.

Inosine-5′-monophosphate dehydrogenase (IMPDH) catalyzes the rate-limiting oxidation of IMP to xanthosine 5′-monophosphate (XMP) in the pathway of guanine nucleotide synthesis and is thus a key player in the regulation of the intracellular purine nucleotide pools of almost every organism^15^. If inhibited, the imbalance between the guanine and adenine nucleotide pools has dramatic consequences for cell proliferation^16^, rendering IMPDHs into a suitable cellular target of drugs widely used to date in chemotherapy as antivirals or as immunosuppressive and antitumor agents^17^. Due to the crucial role in *de novo* nucleotide biosynthesis and the significant clinical relevance, IMPDHs from various species are in the focus of investigations^18,19^.

In 1994, an IMPDH encoding gene was identified in the genome of *T. brucei*^20^. Transmitted by tsetse flies, this protozoan parasite, which still represents a severe human pathogen^21^, infects the blood and the lymphatic system before invading the brain, causing clinical manifestations within weeks or months that are denoted as human African trypanosomiasis (HAT), or sleeping sickness. As the metabolic pathway for the production of purine nucleotides is conspicuously different between parasites and the human hosts, TbIMPDH was proposed to represent a suitable target for anti-trypanosomal drugs to treat *T. brucei* infections, strongly supported by enzymatic differences of TbIMPDH compared to the human counterparts^22^. In particular, the *K*_m_ of TbIMPDH for NAD^+^ is significantly higher than that of mammalian enzymes and indicative of a different NAD^+^ binding mode. Although mammalian cells synthesize purine nucleotides de novo, *T. brucei* is auxotrophic for purines and critically depends on salvage mechanisms, including the IMPDH-catalyzed reaction, to acquire these nucleotides from the extracellular environment. Thus, a TbIMPDH-specific inhibitor could disrupt a biological pathway that is pivotal for the parasite’s life cycle^22^. HAT is still a major health threat in many parts of sub-Saharan Africa, especially in the Democratic Republic of Congo, as chemotherapies are of limited success due to the restricted efficacy and safety of existing drugs, combined with the emergence of drug resistant trypanosome strains^23,24^. Detailed insights into structural differences between human and parasitic IMPDHs may thus provide clues for the development of trypanosome specific inhibitors.

Combining recombinant in cellulo protein crystallization and serial femtosecond crystallography (SFX), we here present the structure of IMPDH from *Trypanosoma brucei*. Needle-shaped crystals isolated from living insect cells with dimensions of up to 70 µm in length and 5 µm in width were delivered to the XFEL beam. Our structural analysis confirmed an IMPDH-characteristic composition consisting of a catalytic TIM barrel^25^ and a regulatory Bateman domain^26^, forming a functional octamer as a quaternary structure. The structure is homologous to bacterial, fungal, and human IMPDHs, however a striking and unexpected feature is the presence of distinct electron density at specific sites in the regulatory Bateman domain that have previously been identified as canonical nucleotide binding sites in other IMPDH proteins^27–29^. This electron density has been interpreted as the occupancy of these positions by one ATP molecule and one GMP molecule, which obviously represent the native nucleotide co-factors. In this context our results strongly support the recently proposed nucleotide-dependent allosteric regulation of eukaryotic IMPDH via the Bateman domain^28,30^. The so far unknown GMP coordination mode observed in this study provides the molecular basis for a compact TbIMPDH octamer conformation that was previously associated with the inactive state of eukaryotic IMPDHs by Buey et al.^28^. However, in fungal IMPDH, these authors observed an essential stabilization of the compact conformation by binding of a third nucleotide to a non-canonical site of the Bateman domain^27,28^ that is not occupied in the TbIMPDH structure presented here. Thus, interfering with individual and most probably specific allosteric regulation might represent a new and innovative starting point to identify novel trypanosome-specific inhibitors, despite the relatively high structural homology of individual IMPDH domains among pro- and eukaryotes. The structural data we present are a starting point in this direction and highlight the potential of intracellular protein crystallization to select specific compounds from the native reservoir present in the cellular environment during intracellular crystal growth. In this context, in cellulo crystallization enables the identification of genuine allosteric co-factors, adding one more important feature to the benefits of intracellular protein crystallization.

## Results

### In cellulo crystallization of TbIMPDH in Sf9 insect cells

**Fig. 1:**
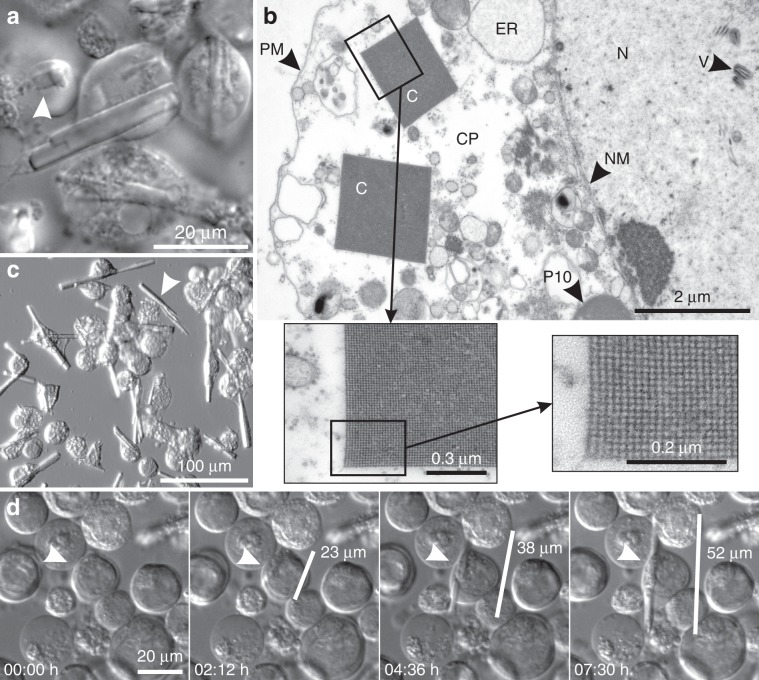
Morphology and growth of TbIMPDH crystals in living insect cells. **a** Differential interference contrast (DIC) image of Sf9 cells 6 days p. i. with recombinant baculovirus containing the gene for TbIMPDH. Needle-shaped crystals, mostly extending the cell-body, are clearly visible. The arrowhead points to a vertical crystal, indicating the rectangular shape of the needles. **b** Transmission electron micrograph of a cross section of two TbIMPDH crystals within the cytoplasm of a Sf9 cell 6 days p. i. showing a clear crystal lattice with large water channels. C TbIMPDH crystal, CP cytoplasm, ER endoplasmic reticulum, N nucleus, NM nuclear membrane, P10 baculoviral P10 protein aggregate, PM plasma membrane, VP - viral particles. **c** Modulation contrast image of Sf9 cells showing multiple TbIMPDH crystals 7 days p. i., located within living cells and free in the surrounding medium (arrowhead). **d** Growth of a TbIMPDH crystal at 5 days p. i. in Sf9 cells taking place over the course of several hours. The crystal grows simultaneously in two dimensions. See also Supplementary Movie 1.

Based on our previous observation that micron-sized crystals of full-length TbIMPDH spontaneously form in insect cells during gene overexpression, comparable to the in cellulo crystallization of TbCatB^31^, we further characterized the intracellular crystallization of TbIMPDH in this study. Approximately 72 h after infection of Sf9 insect cells with recombinant baculovirus that contained the gene encoding full-length *T. brucei* IMPDH, the formation of needle-shaped microstructures with a square base started to be visible by light microscopy (Fig. 1a). TEM investigations clearly showed a crystal lattice with fourfold symmetry and large water channels (Fig. 1b). The crystal growth within the culture continued up to ~day 6 p. i. with individual crystals growing within about 10 h (Fig. 1d, Supplementary Fig. 1, Supplementary Movies 1 and 2). TbIMPDH crystals exhibit maximal dimensions of 70 μm in length and 5 µm in width (Fig. 1b). Most crystals regularly exceed the normal dimensions of Sf9 cells (20–25 µm), without affecting cell viability (Fig. 1c, d, Supplementary Movie 1), as previously observed for TbCatB^31^ and firefly luciferase^32^.

The majority of cells gradually lysed, largely due to the ongoing viral replication process. Individual TbIMPDH crystals floating in the medium or attached to cell remnants were detected by propidium iodide staining, indicating significant crystal stability outside the intact cell (Fig. 1c, Supplementary Fig. 2a). However, within living cells, indications for crystal degradation were observed in later infection stages, sometimes leading to visible crystal disruption (Supplementary Fig. 2, Supplementary Movie 2, Supplementary Movie 3).

**Fig. 2:**
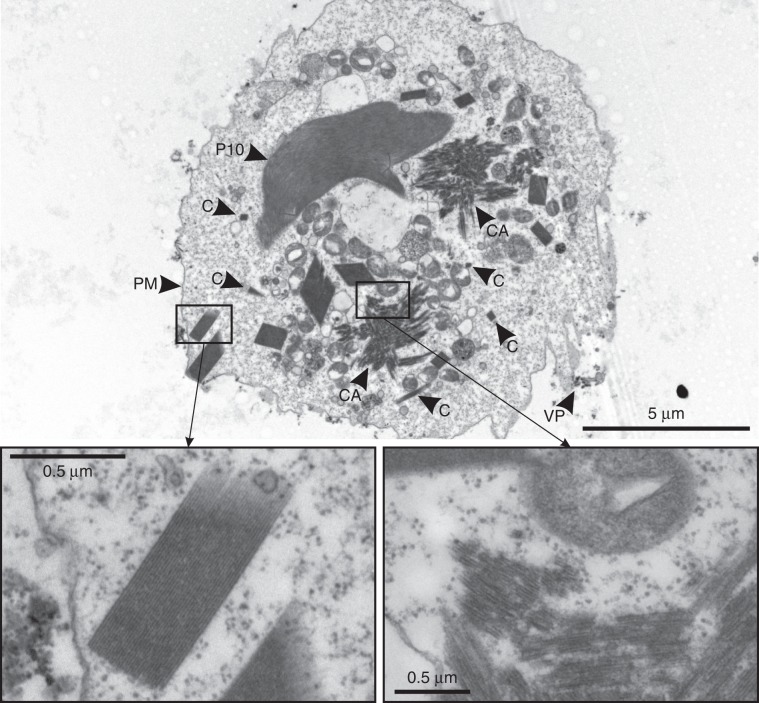
TEM studies of TbIMPDH crystals. Transmission electron micrograph of a Sf9 cell 6 days p.i. showing multiple TbIMPDH crystals with varying dimensions. Sub-micron crystals are indicated by “C”. Signs of infection are clearly visible (baculoviral P10 protein - “P10”, viral particles - “VP”). TbIMPDH not only crystallizes in needle-shaped crystals characterized by a regular crystal lattice (left inset), but also seems to create irregular crystalline assemblies (“CA”, right inset) that display fragmented crystal lattices and spread over several µm within the cytoplasm. PM plasma membrane.

During the progress of infection, the proportion of crystal-containing cells continuously increased until more than 50 % of the population contained one to five visible microcrystals per cell. However, electron microscopy (EM) investigations showed that cells usually contain dozens of small crystals with only a few reaching micrometer size scales, together with additional clusters of microstructures that also contained crystalline lattices to some extend (Fig. 2).

**Fig. 3:**
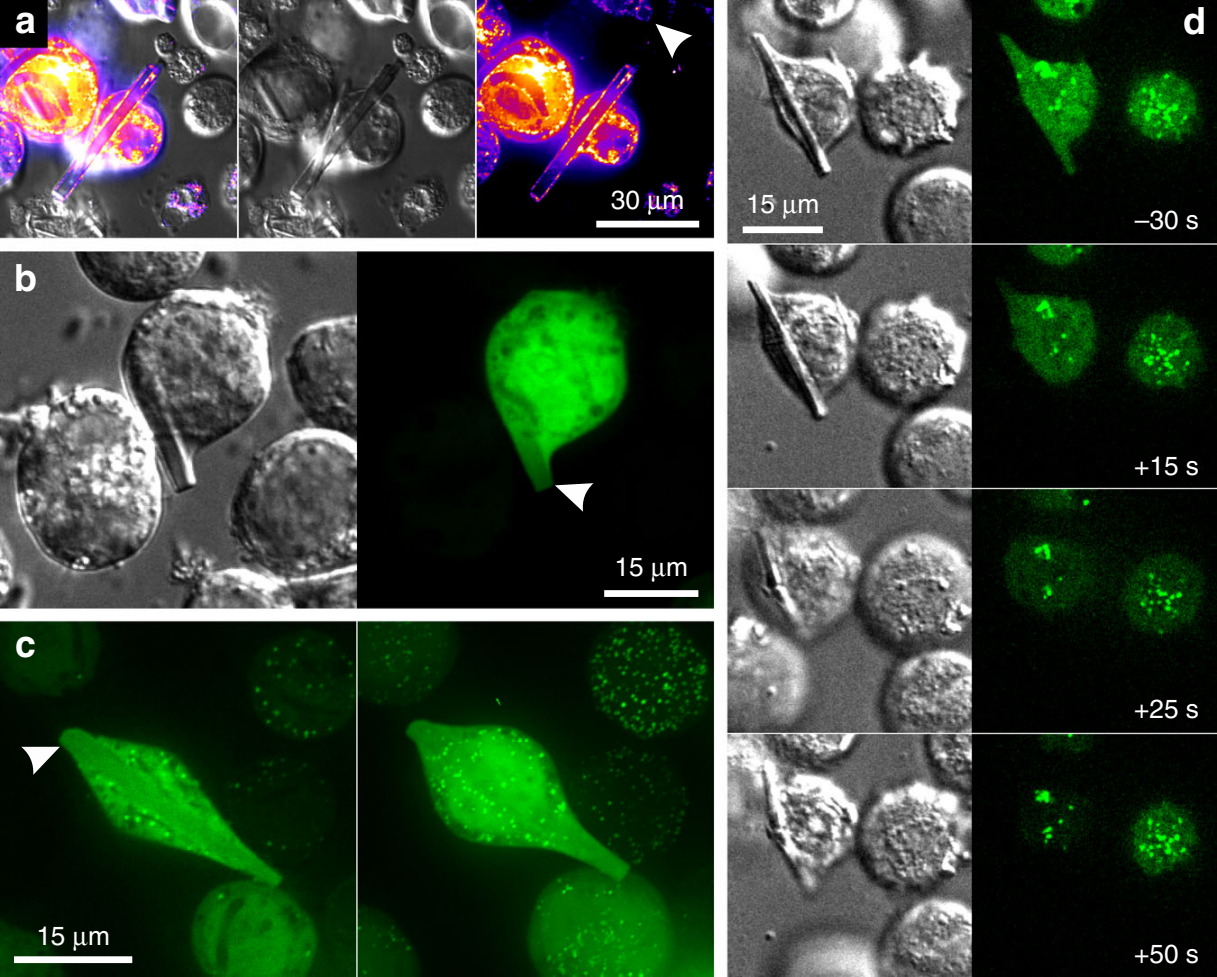
Localization of TbIMPDH crystals within cellular compartments. **a** Co-infection of Sf9 cells with recombinant baculoviruses (rBVs) containing the genes for TbIMPDH and Pex3-mCherry fusion protein 7 days p. i. The Pex3-marker, shown in a “fire” look-up-table for better visibility, labels peroxisomal membranes. The right panel shows that mCherry-fluorescence clearly surrounds TbIMPDH crystals. The white arrowhead points to a crystal vertical in the confocal plane, also surrounded by mCherry fluorescence. Middle panel: DIC image. Left panel: overlay. **b** Co-infection of rBVs containing the genes for TbIMPDH and cytoplasmatic EGFP 6 days p. i. Confocal imaging shows EGFP fluorescence within the crystal volume (white arrowhead), that could result from diffusion of EGFP molecules into channels within the TbIMPDH crystal within the cytosol. **c** Co-infection of rBVs containing the genes for TbIMPDH and peroxisomal EGFP-SKL 7 d. p. i. Left panel: confocal plane showing EGFP fluorescence within the crystal volume, but no enrichment around the crystal demonstrating the cytosolic localization of the crystal. Right panel: maximum projection from a complete Z-stack of the same cell showing many intense point-like structures but no enrichment of EGFP-SKL around the cytosolic crystal. **d** Co-infection of rBVs containing the genes for TbIMPDH and peroxisomal EGFP-SKL 8 days p. i. Right panel: EGFP fluorescence. Left panel: DIC. EGFP-SKL shows cytoplasmatic fluorescence, as well as localized enrichment, identified as peroxisomes. Plasma membrane disruption is visible after treatment with hypotonic buffer at the 0-s-timepoint. EGFP fluorescence is quickly lost from the cytoplasm and the crystal volume, whereas peroxisomes remain undisturbed (see Supplementary Movie 4).

Due to the presence of the native peroxisomal targeting signal 1 (PTS1), a C-terminal ‘SKL’ motif in the TbIMPDH sequence^33^, a peroxisomal origin of the crystals can be expected. Co-infection with a recombinant baculovirus expressing the gene for the peroxisomal membrane marker protein Pex3^32^ confirmed that at least some TbIMPDH in cellulo crystals originate from Sf9 cell peroxisomes (Fig. 3a). However, a close investigation of TEM images did not provide further evidence for a membrane that surrounds the crystals (Figs. 1b and 2). Co-infection with a recombinant baculovirus producing a cytoplasmatic version of enhanced green fluorescent protein (EGFP) shows that the EGFP fluorescence is not excluded from the crystal volume (Fig. 3b), most likely explained by diffusion of EGFP into channels within the crystal lattice. Such an effect can only be explained if crystal growth occurs within the cytoplasm. This is supported by the observation that co-infection with a recombinant baculovirus producing a version of EGFP (EGFP-SKL) that is imported into the peroxisomal lumen did not result in a concentration of EGFP fluorescence around the crystal and thereby within a peroxisomal compartment (Fig. 3c). Furthermore, permeabilization of the plasma membrane with a hypotonic buffer removes EGFP-SKL fluorescence from the crystal volume, but not from dot-like peroxisomal structures (Fig. 3d). Since peroxisomal import from the cytoplasm does not require the cargo protein to be unfolded^34^, growth of in cellulo crystals in both cellular compartments could occur, but most crystals seem to be of cytoplasmatic origin.

### SFX structure determination of TbIMPDH

**Fig. 4:**
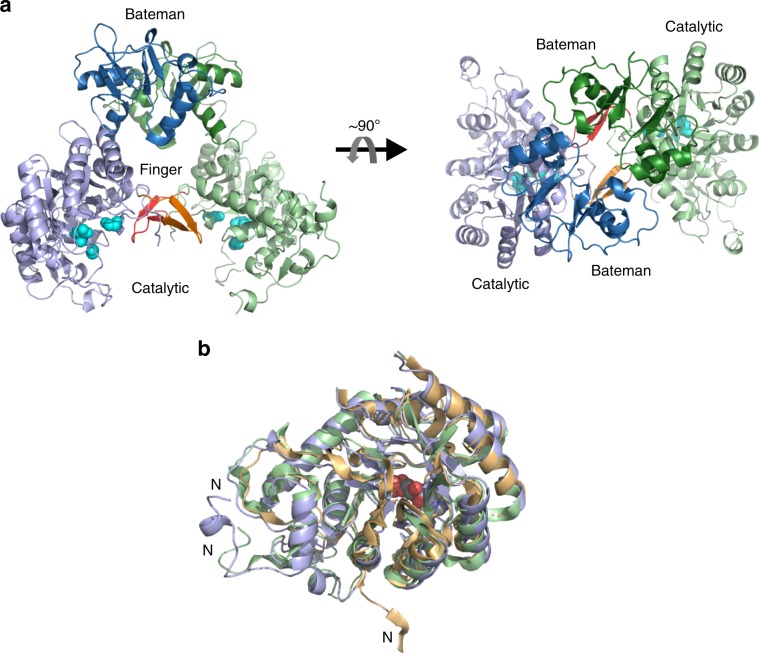
Structure of TbIMPDH and catalytic domain comparison. **a** Side and top view on the two monomers of TbIMPDH (green and blue), each consisting of the catalytic (light colors) and the regulatory Bateman domain (dark colors), that form a dimer in the ASU. The finger domains (red and orange) as well as the residues Asp358, Gly360, and Gly381 that are usually involved in hydrogen bond formation with the ribose and the phosphate moiety of IMP (cyan, in sphere representation) are highlighted. **b** Superposition of the catalytic domain of TbIMPDH (residues 8–120 and 237–514, blue) with corresponding residues of human IMPDH1 (PDB 1JCN, green), in complex with the substrate analog 6-chloropurine riboside 5′-monophosphate (CPR, in sphere representation) to locate the substrate binding site, and of the bacterial IMPDH from *P. aeruginosa* (PDB 1DQW, yellow). N N-terminus.

In cellulo grown crystals of TbIMPDH isolated from baculovirus-infected Sf9 insect cells diffracted up to 2.80 Å resolution, exploiting the 40-fs X-ray pulses of the X-ray free-electron laser (XFEL) Linac Coherent Light Source (LCLS) at the CXI beamline. Applying the SFX technique^4^, 973,000 detector images were collected in 2 h, of which 22,242 were identified as hits (2.3 % hit rate) of TbIMPDH crystals in random orientation. In total, 10,406 of them were successfully indexed and merged into a full dataset (Table 1). Determination of hits and data reduction was performed using Cheetah software^35^. Indexing was done by CrystFEL 0.8.0^36^ using Xgandalf indexing algorithm^37^ allowing multiple crystals indexing per diffraction pattern. Initial detector geometry was taken from optical metrology measurement and then individual quadrants positions and orientation was refined using geoptimiser program^38^. Integration of reflection intensities was done using partialator program from CrystFEL with partiality model ‘unity’. The structure of TbIMPDH was determined by molecular replacement using the coordinates of the human IMPDH isoform 1 (hIMPDH1; monomer A, PDB code 1JCN) as a search model and refined to 2.80 Å resolution. The crystals belonged to the space group P4 2_1_ 2 containing two TbIMPDH monomers in the asymmetric unit (ASU), corresponding to ~75% solvent in the crystal lattice (Fig. 4a). The overall structure superimposed with the hIMPDH1 (PDB code 1JCN) showed an RMSD value of 3.07 Å for 380 equivalent Cα atoms (Supplementary Table 3).

**Table 1:** SFX data collection and refinement statistics.

Data collection	PDB code: 6RFU
XFEL source	LCLS
Wavelength (Å)	1.30
Temperature (K)	291
Space group	P42_1_2
Cell dimensions a, b, c (Å)	209.0, 209.0, 92.0
Number of collected detector frames	973,000
Number of crystal hits (% hit rate)	22,242 (2.3)
Number of indexed crystals (% of hits)	10,406 (47)
Number of unique reflections	50,693
Resolution (Å)	50.63-2.80 (2.87–2.80)
Completeness (%)	100 (100)
Averaged redundancy	98 (52)
Overall I/*σ*(I)	4.43 (1.00)
CC 1/2	0.955 (0.199)
*R*_split_^a^ (%)	21.8 (114.4)
CC^*^	0.988 (0.576)
Refinement	
Resolution (Å)	50.63-2.80 (2.87–2.80)
No. of reflections used in refinement	50,656 (3,567)
*R*_work_/*R*_free_	0.201 (0.346) / 0.230 (0.355)
No. atoms	13,382
Protein	13,203
Nucleotides	157
Water	22
Wilson B factor (Å^2^)	49.4
Overall B factor (Å^2^)	51.9
R.M.S.D	
Bond lengths (Å)	0.005
Bond angles (°)	0.910
Ramachandran plot (%)	
Most favored	96.4
Additionally allowed	3.6
Disallowed	0.0

^a^$$R_{{\mathrm{split}}}{\mathrm{ = }}2^{ - 1/2}\frac{{{\sum} {\left| {I_{{\mathrm{even}}} - I_{{\mathrm{odd}}}} \right|} }}{{\frac{1}{2}{\sum} {\left( {I_{{\mathrm{even}}} - I_{{\mathrm{odd}}}} \right)} }}$$^36^.

Numbers in parenthesis refer to the highest resolution shell.

### The catalytic domain

The TbIMPDH structure closely resembles the typical two-domain fold reported for IMPDHs^39^. The core of the catalytic domain forms a (β/α)_8_ TIM barrel^25^ that is almost superimposable to IMPDH structures of other organisms (Fig. 4b, Supplementary Fig. 3). Significant conformational differences (pairwise RMSD > 2 Å) are limited to the highly flexible N- and C-termini, the finger domain, and to the catalytic Cys325-containing loop (residues 319–335 according to TbIMPDH sequence numbering that will be used throughout the manuscript, Supplementary Fig. 4) which is highly disordered in many of the IMPDH crystal structures^39^, and also not defined by electron density in the TbIMPDH structure. Moreover, no interpretable electron density is observed for TbIMPDH residues 407–438 (chain A) and 409–438 (chain B) that are part of the ‘finger domain’ loop (residues 391–438)^40^ that usually includes a twisted beta sheet and the catalytic flap (residues 419–429), consistent with previous studies^39^. Our observation supports the hypothesis that a high structural flexibility is essential for the catalytic activity of IMPDHs^27,29^. Particularly in the absence of a substrate a significant conformational mobility of the Cys325 loop and the flap region has been reported^41–43^.

### The regulatory Bateman domain

The electron density reveals the entire regulatory domain (residues 114–222), consisting of two tandem repeats of a cystathionine β-synthase (CBS) motif that form a so-called Bateman domain^26^. This motif is located between two short linker sequences (residues 108–113 and residues 223–228) within a loop of the catalytic domain and is well defined in each of the two TbIMPDH monomers in the ASU (Fig. 4a). This is remarkable, since only 10 of the 76 IMPDH structures currently deposited in the PDB include the entire Bateman domain, indicating difficulties to crystallize IMPDH containing this domain by conventional crystallization approaches due to its high degree of disorder^27,44,45^. However, superposition of the TbIMPDH Bateman domain with available structures of complete Bateman domains from *S. pyogenes* (PDB 1ZFJ), *P. aeruginosa* (PDB 4DQW, 6GJV, and 6GK9), *B. anthracis* (PDB 3TSB), *A. gossypii* (PDB 5TC3, 5MCP), and human IMPDH isoform 2 (hIMPDH2; PDB 6I0O, 6I0M) revealed a significant degree of structural homology, despite the relative low sequence identity (Supplementary Fig. 4). Within the standard deviation of the refined structure the regulatory domain of TbIMPDH is superimposable to that of the fungus *A. gossypii* and of hIMPDH2. Besides minor differences at the N- and C-terminal regions significant deviations (pairwise RMSD > 3 Å) to the other three known Bateman domains, all from bacterial IMPDHs, are only found for the short loop connecting the two CBS motifs (residues 176–178) (Supplementary Fig. 5). Moreover, the loop linking the two antiparallel β-strands of CBS1 (residues 144–148) appears to adopt different conformations in eukaryotic and prokaryotic IMPDHs.

### Nucleotide binding in the Bateman domain

**Fig. 5:**
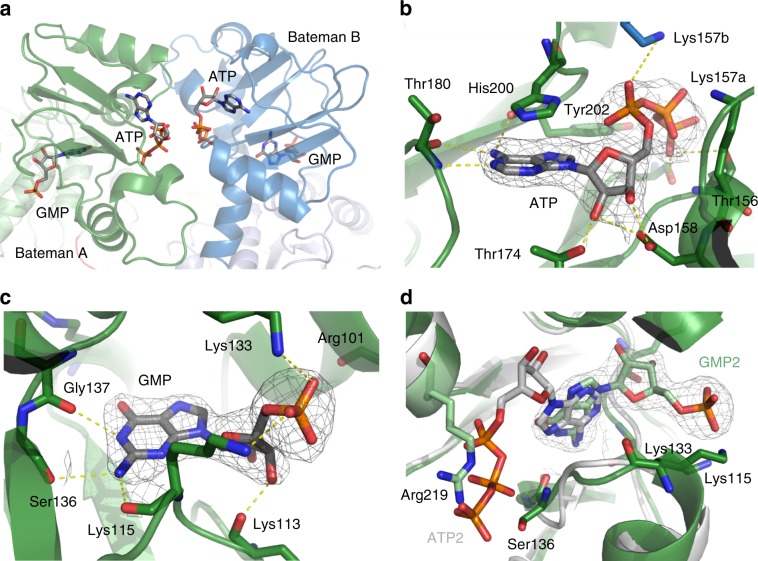
Nucleotide binding to the Bateman domain of TbIMPDH. **a** Electron density detected in two clefts on the surface of the Bateman domain of each monomer in the ASU was assigned to the coordination of one ATP and one GMP molecule in the canonical nucleotide binding sites. **b**, **c** Detailed view of the ATP and GMP binding. The protein is shown as green cartoons and the nucleotides as gray sticks. The side chain of Lys157’ from the neighboring monomer is colored in blue. The ATP and GMP as well as interacting residues are displayed as sticks. Yellow dashes represent hydrogen bonds. **d** Superposition of the second canonical binding site of TbIMPDH-ATP1/GMP2 (green) with that of AgIMPDH-ATP1/ATP2 (gray). Due to the flipped binding mode of GMP2 in TbIMPDH, the side chain of Arg219 occupies the space required for the phosphate groups of ATP2 in AgIMPDH. In **b**–**d**, the displayed 2F_o_F_c_-electron density is countered at 1.0 sigma.

Extra electron density was identified in two clefts on the surface of the Bateman domain that were unambiguously assigned to one ATP molecule and one GMP molecule (Fig. 5a). Although adenine and guanine nucleotide binding sites have recently been predicted and identified within the regulatory Bateman domains of eukaryotic^28^ and prokaryotic IMPDH^29,30,46–48^, this observation is the most interesting, since nucleotides were not added to crystal suspensions during isolation from insect cells, storage, or even in the context of the SFX experiment. Therefore, it can be assumed that native nucleotides from the cytoplasmatic environment of the insect cells have bound to TbIMPDH after gene expression. Nucleotide binding will apparently not change the overall structure of the Bateman domain, as described recently^28,30^. This is confirmed by structural superposition of the nucleotide-free (*S. pyogenes*, *P. aeruginosa*, and *B. anthracis*) Bateman domain with Bateman domains in complex with adenine (*P. aeruginosa* and *A. gossypii*) and guanine (*A. gossypii* and hIMPDH2) nucleotides, as well as with an allosteric inhibitor (*P. aeruginosa*). No significant structural deviations were observed (Supplementary Fig. 5).

The ATP molecule bound to the Bateman domain is located in the previously described ‘first canonical nucleotide binding site’, adopting an extended conformation (Fig. 5b). The presence of an adenine nucleotide is confirmed by the associated and apparent hydrogen bonds, mediating contacts between the backbone carbonyl oxygen atoms of Thr180/Tyr202 and the carbon atom at position 6 of the purine ring, which clearly requires the presence of the hydrogen donating amino group of the adenine. Furthermore, no electron density for an amino group at the C2 atom is observed, excluding a guanine base. The overall interaction signature of ATP corresponds very well with the previously reported coordination of ATP to the canonical sites of the Bateman domains of *A. gossypii*^28^ and of *P. aeruginosa*^29^ IMPDH. Further, the adenine ring is sandwiched by the side chains of residues His200 and Thr174, while in parallel highly conserved hydrogen bonds are established by residue Thr180 to further coordinate the adenine base, by Asp158 and Thr174 to bind the ribose moiety, and by Thr156, Lys157, Tyr202, as well as by Lys157’ (from the adjacent monomer) to coordinate the α- and β-phosphate groups of ATP (Fig. 5b). However, the γ-phosphate group adopts a more extended conformation than observed so far, and is hydrogen bonded to residues Ser136 and Arg219. Slight deviations in this part of the TbIMPDH Bateman domain compared to corresponding parts of the *A. gossypii* and *P. aeruginosa* IMPDHs shift these residues to be in close contact to the γ-phosphate moiety.

The GMP nucleotide occupies the second canonical binding site and is found in a flipped conformation compared to that of the previously reported IMPDH structures in complex with GDP, GTP, and ATP^27,28,30,47^, representing a so far unknown nucleotide binding mode. The purine groups of the different nucleotides consistently occupy the same binding pocket, forming a conserved hydrogen bond pattern involving mainly the carbonyl backbone oxygen atoms of Lys115 and Gly137, and an additional hydrogen bond formed by the Ser136 main chain O atom and the N1 atom of the purine ring in TbIMPDH (Fig. 5c). These interactions crucially depend on the presence of an amino group at the purine C2 atom, supporting the observed binding of the guanine nucleotide, which is also well-defined by the electron density. While the ribose moiety and the phosphate groups of GDP and GTP/ATP usually adopt a compact conformation, which extends inside the Bateman domain, these parts of GMP protrude out of the domain structure into the cleft located between the catalytic and the regulatory domains of TbIMPDH. This conformation is stabilized by interactions involving residues Lys133 (phosphate) in the Bateman domain as well as Lys113 (ribose), Lys115 (phosphate), and Arg101 (phosphate) that are all located in the catalytic domain (Fig. 5c). As a consequence of the observed flipped binding mode, an interaction of the two bound nucleotides in the canonical sites via Mg^2+^ ions, coordinating two ATPs, as described by Labesse et al.^29^ and Buey et al.^27^ for *A. gossypii* and *P. aeruginosa* IMPDHs, is prevented. This missing feature for TbIMPDH might additionally increase the conformational flexibility of the γ-phosphate group of the bound ATP molecule described above. Moreover, the side chain of Arg219 occupies in TbIMPDH the space required for the phosphate groups of GDP and ATP to form a coordination as reported for the IMPDH structures mentioned above (Fig. 5d).

### Relative subdomain orientation

Recent evidences indicate that the activity of the catalytic domain of IMPDH is allosterically controlled by a nucleotide-mediated regulatory switch. While ATP binding maintains certain flexibility of the linker regions connecting the catalytic and the regulatory domains and thus promoting IMPDH activity, guanine nucleotide binding particularly to the second canonical and the recently identified third non-canonical binding site within the Bateman domain is suggested to fix the relative orientation of the rigid subdomains in an inactive conformation^28,30,48^. In GDP-bound *A. gossypii* IMPDH (AgIMPDH-ATP1/GDP2/GDP3 and AgIMPDH-GDP1/GDP2/GDP3), specific interactions are established by the GDP2 and GDP3 nucleotides. Both link the two subdomains with the so-called ‘hinge-bending residues’ within the flexible interconnecting loops, thus supporting the results summarized before^27,28^.

**Fig. 6:**
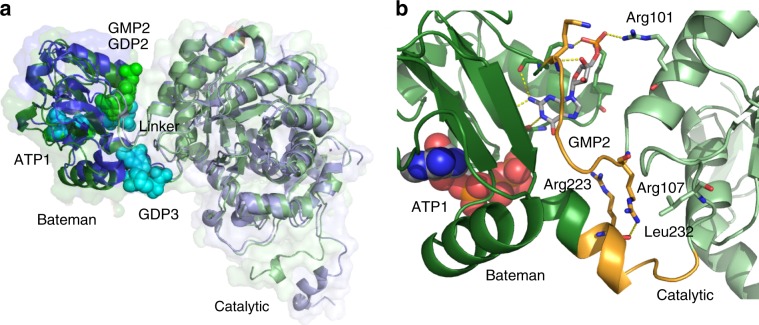
Relative orientation of the catalytic and the regulatory domain. **a** Cartoon and surface representation of superposed monomers A from TbIMPDH-ATP1/GMP2 (green) and from AgIMPDH-ATP1/GDP2/GDP3 (blue, PDB 5TC3). Nucleotide atoms are shown as spheres. Both structures adopt an almost superimposable relative domain orientation. **b** Detailed view of the linker region (orange) between the Bateman (dark green) and the catalytic domains (light green) of TbIMPDH-ATP1/GMP2. The flipped conformation of GMP enables a direct interaction with residue Arg101 in the catalytic domain. Further stabilization is provided by an interaction of Arg107 and Arg223. ATP atoms are shown as spheres, while GMP and the GMP-binding residues as well as interface stabilizing residues are represented as sticks. Key interactions are shown as yellow dashes.

Superposition of the TbIMPDH monomer with corresponding structures of AgIMPDH and hIMPDH2 in complex with different combinations of adenine and guanine nucleotides clearly revealed that the structure of TbIMPDH adopts a specific domain orientation which is highly superimposable to AgIMPDH-GDP1/GDP2/GDP3 (PDB 4Z87) and AgIMPDH-ATP1/GDP2/GDP3 (PDB 5TC3) (Fig. 6a), but also to hIMPDH2-GTP1/GTP2/GTP3 (PDB 6I0O) and hIMPDH2-GDP1/GDP2/GDP3 (PDB 6I0M), consistent with the guanine nucleotide binding in the second canonical binding site of TbIMPDH. Even the linker regions (Asn108 to Lys113 and Arg223 to Pro228 in TbIMPDH) share an almost identical conformation, although the interface stabilization is different. As described above, the flipped conformation of GMP directs the ribose and the phosphate moiety into the cleft between the regulatory and the catalytic domain of TbIMPDH. This enables a direct interaction with the catalytic domain via the side chain of residue Arg101 but prevents interactions of the ribose moiety with the linker residues previously observed in the AgIMPDH-GDP1/GDP2/GDP3 structure (Fig. 6b). Another stabilizing interaction is facilitated between the side chain of Arg107 and the main chain O atom of Arg223 located in the catalytic and the Bateman domain, respectively. This in part compensates for interactions that stabilize the domain orientation in AgIMPDH-GDP1/GDP2/GDP3 but are missing in TbIMPDH due to the vacant third non-canonical nucleotide binding site and a Lys/Leu replacement at position 232, which prevents an ionic interaction with residue Arg107.

In contrast, the relative orientation of the Bateman and catalytic domains in AgIMPDH-ATP1/ATP2 (PDB 5MCP) is significantly different (Supplementary Fig. 6), reflecting the previously reported translation and rotation of the regulatory Bateman domain relative to the catalytic domain as a consequence of ATP binding to the two canonical binding sites^28^. None of the key interactions that stabilize the hinge-bending residues in the ATP-bound state (Glu117-Asn122 and Gln233-Lys461 in AgIMPDH) are observed in our structure.

### Oligomerization of TbIMPDH

**Fig. 7:**
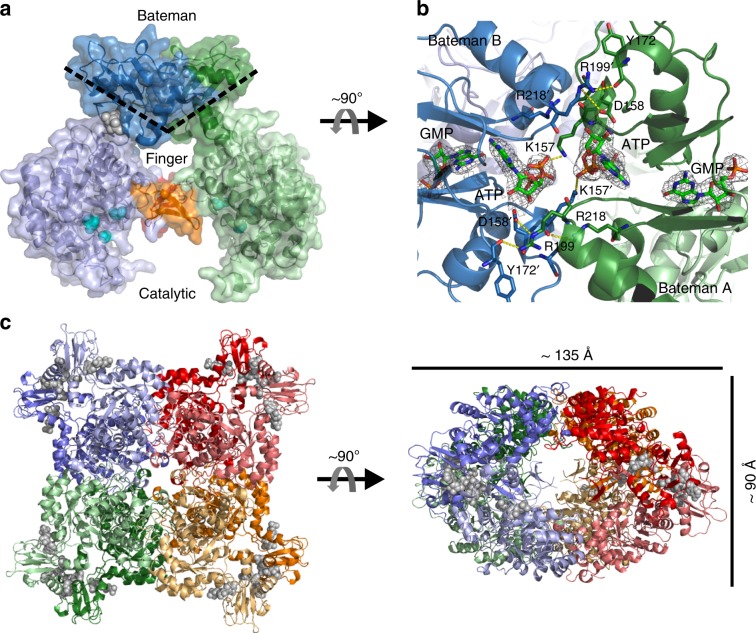
Quaternary structure of TbIMPDH-ATP1/GMP2. **a** Relative orientation of two TbIMPDH monomers located in the ASU, estimated by the approximate angle spanned by the two beta sheets of the CBS motifs in each Bateman domain, as indicated by the dashed line. The finger domains are highlighted (orange/red), the catalytic residues (cyan), and the bound nucleotides (gray) are shown as spheres. **b** Detailed view on the interface formed by the adjacent Bateman domains (green/blue, cartoon representation) within the TbIMPDH dimer. Residues involved in the dimer interaction (yellow dashes) and ATP/GMP molecules are shown as sticks. The displayed 2F_o_F_c_-electron density of ATP and GMP is countered at 1.0 sigma. **c** Cartoon representation of the octamer assembly of TbIMPDH monomers, as observed within the in cellulo crystals and corresponding space group, in different views rotated by 90°. Four dimers are forming an octamer around the 4-fold symmetry axis. The dimeric building blocks observed within the ASU are individually colored. Bound nucleotides are shown in a gray spheres representation.

Two monomers form a dimer in the asymmetric unit (ASU) of the TbIMPDH-ATP1/GMP2 in cellulo grown crystals (Fig. 7a), mainly stabilized by an interface involving the directly facing Bateman domains of both monomers (total BSA 1,104 Å^2^ per monomer) that are in an antiparallel arrangement (Fig. 7b). Key interactions include hydrogen bonds and ionic interactions between the backbone atoms of residues Lys157, Asp160, and Tyr172, as well as the side chains of Asp158, Asp160, Arg199, and Arg218 in a monomer with the respective residues of the adjacent Bateman domain (Supplementary Table 4). Further stabilization of this interface is provided by an inter-domain contact of the ATP β-phosphate group and the side chain of Lys157 (Fig. 7a).

The nucleotide-dependent, relative orientation of the catalytic and the Bateman domain within IMPDH monomers defines a bending angle that reflects the relative orientation of both monomers within the ASU dimer to each other. If guanine nucleotides bind to the second canonical site of the Bateman domain, a more closed angle was previously reported (e.g. 125° in AgIMPDH-GDP1/GDP2/GDP3), compared to the corresponding ATP complexes (e.g. 155° in AgIMPDH-ATP1/ATP2)^28^. This is confirmed for TbIMPDH-ATP1/GMP2, exhibiting a bending angle of ~115° (Fig. 7a). Due to the compact conformation of the TbIMPDH dimer, the finger domains of the adjacent catalytic subunits are in contact. Such finger domain interactions are suggested to be evolutionarily conserved and to be involved in the allosteric activity regulation^27,48^. Nevertheless, an estimation of the impact of these interactions to the overall stability of the TbIMPDH dimer in the ASU was prevented, since some of the residues forming the finger domain are not well defined by the electron density of both TbIMPDH monomers, as also observed in other IMPDH structures.

From the crystal symmetry 4-fold axis, two independent tetramers can be deduced, each characterized by a total BSA of ~15,000 Å² that results from individual interfaces formed by tail-to-tail interactions of the catalytic domains within the dimeric building blocks (BSA 1,105 Å² per monomer) (Supplementary Table 4). Due to a non-crystallographic 2-fold pseudosymmetry, both tetramers generate a TbIMPDH octamer (Fig. 7c) that is classified as a stable biological assembly by the PISA algorithm. Octamers have consistently been observed as quaternary structures for IMPDHs from different organisms, not only inside crystals, but also in solution, suggesting that the octameric assembly indeed exhibits a conserved structural state with high biological relevance^27,29^.

The more compact conformation of the TbIMPDH-ATP1/GMP2 octamer, characterized by an approximated volume of 135 × 135 × 90 Å^3^, closely resembles equivalent oligomers previously described for apo-states of bacterial IMPDHs from *P. aeruginosa*^29,48^, *S. pyogenes*^40^, and *B. anthracis*^45^. However, also octamers of eukaryotic IMPDH from *A. gossypii* and hIMPDH2 share this compact conformation, but only if guanine nucleotides occupy the second canonical and the non-canonical binding sites of the Bateman domain^27,28,30,48^ (Supplementary Fig. 7, Supplementary Table 5). In contrast, ATP coordination induces a remarkably different and significantly extended octamer conformation, mainly attributed to the impact of ATP on the relative subdomain orientation within a monomer that mainly affects the interface of the dimeric building blocks^28,29^.

## Discussion

The room-temperature X-ray structure of TbIMPDH-ATP1/GMP2 presented in this study represents the third successful application of crystals grown in living insect cells to obtain high-resolution structural information of a recombinant protein, next to TbCatB^11^ and CPV1 polyhedrin^49^, providing additional evidence that in cellulo crystallized proteins can be used as suitable targets for structural biology. TbCatB and TbIMPDH do not share any similarities other than the source organism. However, within 3–6 days after baculovirus infection of the insect cells, the intracellular crystallization process consistently led to the formation of needle-shaped crystals comparable to those observed for firefly luciferase and reoviral GFP-µNS proteins^32^, indicating common principles of a more general crystallization mechanism that can be exploited for other proteins as well. A prolific interplay between high local protein concentrations and the intrinsic crystallization tendency of the target proteins at specific cellular conditions has already been suggested to favor in cellulo crystal growth^7,10^. Similar to GFP-µNS, the cytoplasm of insect cells promote TbIMPDH crystallization in this study, while TbCatB crystals originate from the rough endoplasmic reticulum and luciferase from the peroxisomes^31,32^. Thus, the different environmental conditions provided by the individual cellular organelles might represent the basis to develop a more systematic in cellulo crystallization screening approach for recombinant proteins in the future.

The spontaneous in cellulo crystallization of TbIMPDH-ATP1/GMP2 was an unexpected event. Originally, our aim was to produce the soluble protein in insect cells, followed by affinity chromatography purification and conventional crystallization screening. Thus, we used a gene construct that encoded a six-fold His-tag followed by a TEV protease cleavage site N-terminal to the full-length TbIMPDH sequence. However, the artificial N-terminus, which is not defined by electron density and thus considered to be highly flexible, does apparently not impact the structure of TbIMPDH. This is indicated by the significant structural homology compared to IMPDH structures from other organisms.

Our results highlight the advantages of protein crystallization during heterologous gene expression in living cells under quasi-native conditions. In addition to the large-scale production of crystals suitable for SFX, including post-translationally modified proteins in their biologically functional form as already shown for fully glycosylated TbCatB^11^, the need for time-consuming optimization of protein purification protocols and extensive crystal screening experiments is eliminated. The latter is particularly important for proteins that are not obtained in a well-folded native state after recombinant gene expression, or that are difficult to crystallize by applying conventional in vitro methods, as observed for TbIMPDH. Trials to obtain soluble proteins by gene overexpression in *E. coli* and in insect cells or by re-solving isolated in cellulo grown crystals failed, since TbIMPDH immediately aggregated and precipitated due to mis- and unfolding, preventing an additional biochemical and/or biophysical characterization of TbIMPDH in solution so far. Moreover, the frequently observed disordered Bateman domain is most probably preventing the in vitro crystallization of full-length IMPDH proteins in most cases^39,44^, as reflected by the 10 full-length structures out of 76 IMPDH structures currently available in the PDB.

Most remarkably, the TbIMPDH-ATP1/GMP2 structure presented here further confirms the tremendous potential of in cellulo crystallization by highlighting opportunities to identify native co-factors, which are present in the highly versatile natural reservoir of compounds within the living cell. The well-defined electron density within the first and second canonical nucleotide binding site of the TbIMPDH’s Bateman domain and the unique interaction signature allow for the unambiguous identification of ATP and GMP nucleotides, respectively.

CBS motifs are well-known to act as functional adenosine nucleotide binding motifs in several proteins^39,50^, as described for the Bateman domain of prokaryotic^29,46^ and eukaryotic IMPDHs^28,30^. However, the trypanosomal enzyme represents, to our knowledge, the third example of an IMPDH that specifically binds guanine nucleotides through its regulatory domain, in addition to fungal AgIMPDH^27,28^ and hIMPDH2^30^, but the first IMPDH that shows GMP coordination, in contrast to GDP in AgIMPDH and GDP or GTP in hIMPDH2. This is of particular interest, since GDP has recently been proposed to act as a natural negative effector in a species-dependent mechanism for allosteric activity regulation in IMPDH, shedding light on structural details required for the communication between the Bateman and catalytic domain, which till now remained unclear^27,28,30,46^. Following the proposed model, it can be concluded that Bateman domains of class-I bacterial IMPDHs require the coordination of two ATP molecules in the canonical binding sites to achieve full catalytic activity, whereas class-II bacterial and eukaryotic IMPDHs are not affected by ATP binding^28,29,46^. However, if ATP and GDP compete for Bateman domain binding, as analyzed only for fungal AgIMPDH so far, GDP replaces ATP from the second canonical site and additionally binds to a third site, recently identified as a non-canonical motif^27,28^. Both GDP molecules are essentially involved in an interaction network at the subdomain interface that restricts the flexibility within the AgIMPDH monomer, locking the biologically relevant octamer in a more compact conformation. This enables direct interactions of the finger domains that form a small central oligomeric interface, resulting in less flexible catalytic domains with a compromised catalytic activity^28^. Since a comparable rigidification of the catalytic domain has recently been reported for inactive *P. aeruginosa* IMPDH in the apo state and in complex with an allosteric inhibitor (F2K) that partly occupies the first canonical nucleotide binding site in the Bateman domain, the allosteric modulation of the catalytic activity of IMPDH appears to be conserved on the structural level^48^.

The monomer and octamer conformation of native co-factor bound TbIMPDH-ATP1/GMP2 observed in this study largely coincides with that of previously reported AgIMPDH-ATP1/GDP2/GDP3 and AgIMPDH-GDP1/GDP2/GDP3, but also of the very recently elucidated GDP1/GDP2/GDP3 and GTP1/GTP2/GTP3 complexes of hIMPDH2, all formed by incubation of the purified enzyme with a large excess of these selected nucleotides^28,30^. The overall agreement of the artificially and intracellularly formed complexes strongly supports the important and specific biological relevance of the adenine/guanine nucleotide-dependent regulatory mechanism proposed by Buey et al. This is further highlighted by the recently deposited structure of *T. brucei* GMP reductase (GMPR, PDB 5X8O) in complex with GTP, which shares the same overall fold as TbIMPDH (Supplementary Table 3 and Supplementary Fig. 7). The GTP coordination at the second canonical binding site of the Bateman domain of TbGMPR is again associated with a more close bending angle in the dimer and thus with the formation of a compact TbGMPR oligomer structure, highly consistent with IMPDHs harboring a guanine nucleotide at the second site (Supplementary Fig. 8). However, instead of GDP or GTP, GMP occupies the second canonical site in TbIMPDH, exhibiting a flipped, so far unknown nucleotide coordination mode for Bateman domains. This enables the formation of direct interactions with the catalytic domain, resulting in a stabilization of the compact IMPDH oligomer conformation. Based on enzyme kinetic studies GMP was proposed to act as a competitive inhibitor for IMPDHs, only binding to the active site in the catalytic domain by resembling the conformation of the substrate^27^. In AgIMPDH, GMP induced a slight compaction of the oligomeric assembly in solution but attempts to crystallize full-length AgIMPDH in complex with GMP were unsuccessful, preventing a more detailed structural investigation so far.

Taking into account that TbIMPDH-ATP1/GMP2 crystals have been subjected to extensive washing steps during the isolation process, followed by storage in PBS buffer until the SFX experiment, a high-affinity binding of ATP and GMP to the canonical sites is confirmed and required to prevent dissociation. This also shows a most specific selection of the genuine allosteric co-factors from a highly heterogeneous nucleotide pool in the living cell. Considering the intracellular GMP (60 ± 40 µM) and ATP concentrations (from 0.5 to 10 mM)^51^, a predominant saturation of the canonical sites with ATP might be expected. However, the TbIMPDH structure clearly reveals guanine nucleotide specificity for the second canonical site in a quasi-physiological environment of the enzyme.

Thus, an interpretation could be that the conformational regulatory switch might indeed be unidirectional within the cell, i.e., guanine nucleotides can inhibit ATP-induced IMPDH activation, but ATP cannot reverse guanine nucleotide-dependent inhibition. Moreover, the absence of additional guanine nucleotides in the non-canonical binding site and in the active center of the catalytic domain of TbIMPDH indicates a significantly reduced binding affinity to these motifs, which might enable nucleotide dissociation during preparation of the in cellulo crystal suspension. On the other hand, an artificial character of corresponding observations reported in AgIMPDH due to low-affinity guanidine nucleotide binding in the presence of up to 10 mM GMP^27^, a concentration range which represents up to a 200-fold excess compared to the intracellular GMP concentrations^51^, needs to be considered. Since equivalently high GDP concentrations have also been used to form the corresponding AgIMPDH complexes, these artificial conditions might have also affected the GDP coordination particularly for the second canonical site, which needs to be investigated in terms of future studies.

In summary, the combination of in cellulo crystallization and SFX data collection at an XFEL source enabled the elucidation of an IMPDH structure from the parasite *T. brucei*, that was not accessible applying conventional approaches so far, and allowed the identification of two native nucleotide co-factors, ATP and GMP, bound at the canonical sites of the regulatory Bateman domain. In this context our results strongly support the recently proposed nucleotide-dependent allosteric activity regulation in eukaryotic IMPDHs, complemented by highlighting a novel GMP coordination mode. However, future studies of other IMPDHs need to address the question if the specific GMP binding to the regulatory Bateman domain is unique for TbIMPDH, or if this nucleotide is of relevance for IMDPHs in general. Moreover, and despite the overall structural homology of the individual domains of TbIMPDH to other IMPDHs from pro- and eukaryotes, the detected individual and specific allosteric activity regulation might represent a suitable target for a novel trypanosome-specific inhibitor design, now enabled by the elucidation of the important structural framework of this enzyme. Our investigations confirm as well that in cellulo crystallization in combination with serial crystallography at XFEL sources offers exciting new possibilities and features in structural biology and structure-based drug discovery.

## Methods

### Cloning

The gene coding for *T. brucei* IMPDH (Accession number M97794) was amplified by PCR using primers 5′-GGATCCATGGAAAACACCAACCTACGC-3′ (sense), 5′-GCAAGCTTAGAGCTTCGAGGCAAAGAG-3′ (antisense) and AccuPrime™ Taq DNA polymerase (Invitrogen) with trypanosome cDNA according to the manufacturer’s instructions. After subcloning (TOPO-TA cloning kit, Invitrogen) into XL1-Blue competent *E. coli* cells (Stratagene), plasmid DNA purification (QIAprep spin miniprep kit, Qiagen), and digestion with BamHI and HindIII, the extracted gel fragment (QIAquick gel extraction kit, Qiagen) was cloned into pFastBacHTb expression plasmid (Invitrogen) that provided an additional gene sequence encoding a sixfold His-tag and a TEV protease cleavage site fused to the N-terminus of the TbIMPDH gene. The construct was sequenced and transformed into DH10Bac competent *E. coli* cells (Invitrogen) according to the manufacturer’s instructions.

Recombinant Bacmid-DNA was purified using the QIAprep spin miniprep kit (Qiagen) and subsequently used for PCR-analysis of the cloned sequence. Correctness of the PCR products was verified by sequencing. Bacmid-DNA was then used for lipofection with Sf9 insect cells grown in EX-CELL 420 serum-free medium at 27 °C to generate recombinant virus stock according to the Bac-to-Bac-manual (Invitrogen). This stock was used to generate a high titer virus stock for further infections (Titer: 1 × 10^8^ pfu/mL).

The construction of the recombinant baculoviruses coding for Pex3-mCherry and Pex26-mCherry has previously been described^32^. For baculovirus–driven expression of the organelle markers EGFP and EGFP-SKL, the EGFP gene was amplified by PCR using primers 5′- GATCGGATCCATCATGGTGAGCAAGGGCGAG-3′ (sense), 5′- GATCCTCGAGTTACTTGTACAGCTCGTCCATGC-3′ (antisense, EGFP) or 5′- GATCCTCGAGTTACAGCTTGGACTTGTACAGCTCGTCCATGC-3′ (antisense, EGFP-SKL) using Phusion DNA polymerase (Thermo Fisher Scientific) according to the manufacturer’s instructions. The amplicons were subcloned into pFastBac1 vector using BamHI and XhoI restriction enzymes and sequenced. The construction of recombinant baculoviruses and subsequent generation of high-titer virus stocks were performed as outlined above.

### Determination of the viral titer

The dilution assay was used to identify the titer of the viral P3 stocks. In a 96-well plate 0.2 mL of a 5 × 10^4^ cells/mL suspension of Sf9 cells in EX-CELL 420 serum-free medium (Sigma) were added in each well and incubated for 1 h to let cells attach to the bottom. Then a serial dilution (10^−3^–10^−8^) of the virus solution with medium was prepared. For each virus dilution seven wells of the plate were infected with 10 µL of virus dilution at a time. Infection with medium instead of virus dilution served as a negative control. After 5 days at 27 °C the wells with cells that showed signs of infection were counted and the virus titer was calculated using the TCID_50_ (tissue culture infectious dose 50).

### Sf9 insect cell culture

Sf9 insect cells were adapted prior to growth in suspension or monolayer to serum-free EX-CELL 420 insect cell culture medium (Sigma) at 27 °C. Suspension culture cells were usually seeded at 0.5–1 × 10^6^ cells/mL in a total volume of 25 mL in an upright standing 75 cm^2^ disposable T-flask. Cells were exponentially grown, incubated in a controlled shaker at 27 °C and 100 rpm. Cell density was counted daily. When the density reached 4 × 10^6^ cells/mL cells were split.

### Production and isolation of TbIMPDH crystals

Recombinant virus stock was used to infect a suspension culture of Sf9 insect cells seeded at 1 × 10^6^ cells/mL, grown in serum-free medium at 27 °C with a multiplicity of infection (MOI) of 0.1 pfu/cell. After 120–168 h (determined by visual inspection of crystal amount by light microscopy) the cells were harvested by centrifugation at 1000 rcf for 5 min, and lysed by resuspension of the pellet in RIPA buffer (purchased from Alfa Aesar). Crystals were subsequently pelleted at 5000 rcf for 5 min, washed, and stored in phosphate buffered saline (PBS).

### Transmission electron microscopy

For transmission electron microscopy (TEM), infected insect cells of a 3.9-cm^2^ confluent monolayer culture were fixed using 2% (v/v) glutaraldehyde and 0.6% (w/v) paraformaldehyde in 60 mM sodium cacodylate buffer containing 2.7 mM CaCl_2_ for 4 h at 4 °C. After washing for 30 min in 120 mM sodium cacodylate buffer, cells were postfixed in 2% osmium tetroxide (w/v) and washed two times for 15 min in 120 mM sodium cacodylate buffer. Dehydration in ethanol, clearing in propylene oxide, embedding in Araldite epoxy resin, and sectioning was performed according to standard procedures. Sections were stained in 0.5% (w/v) uranyl acetate and 3% (w/v) lead citrate. TEM was performed using a JEOL JEM-1011.

### Optical microscopy and live cell imaging

Sf9 cells were plated on 25 mm glass coverslips to 50% confluency and incubated for 1 h at 27 °C. After infection with recombinant baculovirus and incubation for 4 days, adherent cells were imaged using a laser confocal spinning disk microscope system based on a Nikon Ti-Eclipse microscope equipped with a Yokogawa CSU-X1 unit and an Andor iXon + EMCCD camera. The microscope was fitted with ×40, 1.30 NA and ×100, 1.49 NA objectives. Image acquisition was controlled with Andor Bioimaging software (Andor IQ2.1). Protein crystal growth and dynamics in living Sf9 cells were recorded by time-lapse microscopy using differential interference contrast (DIC) optics. Staining of lysosomes was performed with LysoTracker Deep Red (Life Technologies). Cells were mounted on the live cell microscope and stained with 40 nM LysoTracker in culture medium for 15 min at 26 °C. Fluorescence was elicited with 640 nm laser light and imaged with appropriate filter settings. For co-localization of TbIMPDH crystals with fluorescent organelle marker protein chimeras, cells were plated at 50% confluence and co-infected with identical amounts of recombinant baculovirus stocks coding for TbIMPDH and the marker protein. EGFP fluorescence was excited with 488 nm and mCherry with 561 nm laser light and imaged with appropriate filter settings. Propidium iodide staining was performed directly on the microscope stage using a working concentration of 500 ng/mL in medium and incubation for 20 min at 26 °C. Fluorescence was excited using a 561-nm laser. Coloring and overlays of the original 14-bit grayscale images was done using ImageJ software with included “green” and “fire” lookup-tables.

### Serial femtosecond crystallography

Diffraction data of in cellulo grown TbIMPDH crystals were collected applying the SFX technique at the LCLS CXI instrument at the SLAC National Accelerator Laboratory in Stanford, CA, USA^52^. The XFEL generated intense monochromatic X-ray pulses of 40 fs duration with 4–8 × 10^11^ photons per pulse and a wavelength of 1.299 Å (9.4 keV) that were focused to ~4 µm beam diameter at the interaction point using beryllium compound refractive lenses, corresponding to a peak power density in excess of 10^17^ W cm^−2^ at the sample. The electron and photon beam parameters are summarized in Supplementary Table 1.

A suspension of purified in cellulo grown TbIMPDH crystals adjusted to approximately 1 × 10^9^ crystals per mL in PBS were injected into the XFEL beam using a liquid jet^53^ focused to a diameter of ~4 μm at a flow rate between 25 and 30 μl min^−1^. A rotating syringe device^54^ was used to avoid settling of the crystals during sample storage and delivery. The position of the X-ray beam was adjusted to intersect the continuous liquid column, before the Rayleigh break-up of the jet into drops. Single pulse diffraction patterns from randomly oriented TbIMPDH crystals were recorded at 120 Hz repetition rate on a Cornell-SLAC Pixel Array Detector (CSPAD)^55,56^ that was positioned at distances of 95 mm and 105 mm from the interaction region.

Peak detection and subsequent hit finding were performed using the Cheetah software package^35^. Peakfinder8 algorithm was used with the following parameters: minimum SNR of 7, 50 ADC threshold, and at least 2 pixels per Bragg peak. Patterns that contained more than 20 detected Bragg peaks were deemed a hit. A total of 22,242 frames out of the 973,000 recorded detector patterns were identified as hits, representing a ‘hit rate’ of 2.3%. These patterns were then passed to the CrystFEL software package^36^ for indexing and averaging, applying the unit cell parameters of in cellulo grown TbIMPDH crystals. Option –multi was used allowing to index multiple crystals per diffraction pattern. A total of 10,406 indexed crystals (47% indexing rate) yielded a complete set of structure factors from 50.63 to 2.80 Å resolution merged from 50,693 reflections. Data collection statistics are summarized in Table 1 and in Supplementary Table 2.

### Structure determination

SFX data were phased by molecular replacement using Phaser-MR^57^ and the coordinates of monomer A of hIMPDH1 (PDB 1JCN) as a search model, which exhibits a sequence identity of 52.5% to the *T. brucei* IMPDH. During different stages of model building and refinement using COOT^58^ and phenix.refine^59^, respectively, two molecules of ATP and two molecules of GMP were modeled in difference electron densities in the AU. The structure of IMPDH was refined at a resolution of 2.80 Å with a final R-factor of 20.1% and an *R*_free_ = 23.0%. Refinement statistics are summarized in Table 1. All illustrations were prepared using PyMol v1.3 (DeLano Scientific; http://www.pymol.org). Structural superpositions were performed with the program superpose^60^.

## Supplementary information

Supplementary Information

Description of Additional Supplementary Files

Supplementary Movie 1

Supplementary Movie 2

Supplementary Movie 3

Supplementary Movie 4

## Data Availability

Coordinates and structure factors have been deposited in the Protein Data Bank with accession code 6RFU. Other data are available from the corresponding author upon reasonable request.

## References

[1] Martin-Garcia JM, Conrad CE, Coe J, Roy-Chowdhury S, Fromme P (2016). Serial femtosecond crystallography: a revolution in structural biology. Arch. Biochem. Biophys..

[2] Schlichting I (2015). Serial femtosecond crystallography: the first five years. IUCrJ.

[3] Standfuss J, Spence J (2017). Serial crystallography at synchrotrons and X-ray lasers. IUCrJ.

[4] Chapman HN (2011). Femtosecond X-ray protein nanocrystallography. Nature.

[5] Nass K (2015). Indications of radiation damage in ferredoxin microcrystals using high-intensity X-FEL beams. J. Synchrotron Radiat..

[6] Nass K (2019). Radiation damage in protein crystallography at X-ray free-electron lasers. Acta Crystallogr. Sect. D. Struct. Biol..

[7] Schönherr R, Rudolph JM, Redecke L (2018). Protein crystallization in living cells. Biol. Chem..

[8] Doye JPK, Poon WCK (2006). Protein crystallization in vivo. Curr. Opin. Colloid Interface Sci..

[9] Coulibaly F (2007). The molecular organization of cypovirus polyhedra. Nature.

[10] Duszenko M (2015). In vivo protein crystallization in combination with highly brilliant radiation sources offers novel opportunities for the structural analysis of post-translationally modified eukaryotic proteins. Acta Crystallogr. F Struct. Biol. Commun..

[11] Redecke L (2013). Natively inhibited Trypanosoma brucei cathepsin B structure determined by using an X-ray laser. Science.

[12] Tsutsui H (2015). A diffraction-quality protein crystal processed as an autophagic cargo. Mol. Cell.

[13] Baskaran Y (2015). An in cellulo-derived structure of PAK4 in complex with its inhibitor Inka1. Nat. Commun..

[14] Colletier J-P (2016). De novo phasing with X-ray laser reveals mosquito larvicide BinAB structure. Nature.

[15] Wang W, Hedstrom L (1997). Kinetic mechanism of human inosine 5’-monophosphate dehydrogenase type II: random addition of substrates and ordered release of products. Biochemistry.

[16] Jayaram HN, Cooney DA, Grusch M, Krupitza G (1999). Consequences of IMP dehydrogenase inhibition, and its relationship to cancer and apoptosis. Curr. Med. Chem..

[17] Braun-Sand SB, Peetz M (2010). Inosine monophosphate dehydrogenase as a target for antiviral, anticancer, antimicrobial and immunosuppressive therapeutics. Future Med. Chem..

[18] Shah, C. P. & Kharkar, P. S. Inosine 5'-monophosphate dehydrogenase inhibitors as antimicrobial agents: recent progress and future perspectives. *Future Med. Chem*. **7**, 1415–1429 (2015). 10.4155/fmc.15.72 26230881

[19] Sarwono AEY (2019). Repurposing existing drugs: identification of irreversible IMPDH inhibitors by high-throughput screening. J. Enzyme Inhib. Med. Chem..

[20] Wilson K, Berens RL, Sifri CD, Ullman B (1994). Amplification of the inosinate dehydrogenase gene in Trypanosoma brucei gambiense due to an increase in chromosome copy number. J. Biol. Chem..

[21] Tiberti N, Sanchez J-C (2018). Sleeping sickness in the ‘Omics era. Proteom. Clin. Appl..

[22] Bessho T (2013). Characterization of the novel Trypanosoma brucei inosine 5’-monophosphate dehydrogenase. Parasitology.

[23] Barrett MP, Boykin DW, Brun R, Tidwell RR (2007). Human African trypanosomiasis: pharmacological re-engagement with a neglected disease. Br. J. Pharmacol..

[24] Alsford S (2012). High-throughput decoding of antitrypanosomal drug efficacy and resistance. Nature.

[25] Nagano N, Orengo CA, Thornton JM (2002). One fold with many functions: the evolutionary relationships between TIM barrel families based on their sequences, structures and functions. J. Mol. Biol..

[26] Bateman A (1997). The structure of a domain common to archaebacteria and the homocystinuria disease protein. Trends Biochem. Sci..

[27] Buey, R. M. et al. Guanine nucleotide binding to the Bateman domain mediates the allosteric inhibition of eukaryotic IMP dehydrogenases. *Nat. Commun*. **6**, 8923 (2015). 10.1038/ncomms9923 PMC4660370 26558346

[28] Buey RM (2017). A nucleotide-controlled conformational switch modulates the activity of eukaryotic IMP dehydrogenases. Sci. Rep..

[29] Labesse G (2013). MgATP regulates allostery and fiber formation in IMPDHs. Structure.

[30] Fernández-Justel D (2019). A nucleotide-dependent conformational switch controls the polymerization of human imp dehydrogenases to modulate their catalytic activity. J. Mol. Biol..

[31] Koopmann R (2012). In vivo protein crystallization opens new routes in structural biology. Nat. Methods.

[32] Schönherr R (2015). Real-time investigation of dynamic protein crystallization in living cells. Struct. Dyn..

[33] Keller Ga, Gould S, Deluca M, Subramani S (1987). Firefly luciferase is targeted to peroxisomes in mammalian cells. Proc. Natl Acad. Sci. USA.

[34] Francisco T (2017). Protein transport into peroxisomes: Knowns and unknowns. BioEssays.

[35] Barty A (2014). Cheetah: software for high-throughput reduction and analysis of serial femtosecond X-ray diffraction data. J. Appl. Crystallogr.

[36] White TA (2012). CrystFEL: a software suite for snapshot serial crystallography. J. Appl. Crystallogr.

[37] Gevorkov Y (2019). XGANDALF - extended gradient descent algorithm for lattice finding. Acta Crystallogr. A.

[38] Yefanov O (2015). Accurate determination of segmented X-ray detector geometry. Opt. Express.

[39] Hedstrom L (2009). IMP dehydrogenase: structure, mechanism, and inhibition. Chem. Rev..

[40] Zhang R (1999). Characteristics and crystal structure of bacterial inosine-5’-monophosphate dehydrogenase. Biochemistry.

[41] Prosise GL, Wu JZ, Luecke H (2002). Crystal structure of Tritrichomonas foetus inosine monophosphate dehydrogenase in complex with the inhibitor ribavirin monophosphate reveals a catalysis-dependent ion-binding site. J. Biol. Chem..

[42] Morrow CA (2012). De novo GTP biosynthesis is critical for virulence of the fungal pathogen Cryptococcus neoformans. PLoS Pathog..

[43] Rao VA, Shepherd SM, Owen R, Hunter WN (2013). Structure of Pseudomonas aeruginosa inosine 5’-monophosphate dehydrogenase. Acta Crystallogr. Sect. F. Struct. Biol. Cryst. Commun..

[44] Buey RM, Ledesma-Amaro R, Balsera M, de Pereda JM, Revuelta JL (2015). Increased riboflavin production by manipulation of inosine 5’-monophosphate dehydrogenase in Ashbya gossypii. Appl. Microbiol. Biotechnol..

[45] Makowska-Grzyska M (2012). Bacillus anthracis inosine 5’-monophosphate dehydrogenase in action: the first bacterial series of structures of phosphate ion-, substrate-, and product-bound complexes. Biochemistry.

[46] Alexandre T, Raynal B, Rayna B, Munier-Lehmann H (2015). Two classes of bacterial IMPDHs according to their quaternary structures and catalytic properties. PLoS ONE.

[47] Labesse G, Alexandre T, Gelin M, Haouz A, Munier-Lehmann H (2015). Crystallographic studies of two variants of Pseudomonas aeruginosa IMPDH with impaired allosteric regulation. Acta Crystallogr. D. Biol. Crystallogr..

[48] Alexandre T (2019). First-in-class allosteric inhibitors of bacterial IMPDHs. Eur. J. Med. Chem..

[49] Boudes M, Garriga D, Fryga A, Caradoc-Davies T, Coulibaly F (2016). A pipeline for structure determination of in vivo-grown crystals using in cellulo diffraction. Acta Crystallogr. Sect. D. Struct. Biol..

[50] Ereño-Orbea J, Oyenarte I, Martínez-Cruz LA (2013). CBS domains: ligand binding sites and conformational variability. Arch. Biochem. Biophys..

[51] Traut TW (1994). Physiological concentrations of purines and pyrimidines. Mol. Cell. Biochem..

[52] Emma P (2010). First lasing and operation of an ångstrom-wavelength free-electron laser. Nat. Photonics.

[53] Weierstall U, Spence JCH, Doak RB (2012). Injector for scattering measurements on fully solvated biospecies. Rev. Sci. Instrum..

[54] Lomb L (2012). An anti-settling sample delivery instrument for serial femtosecond crystallography. J. Appl. Crystallogr.

[55] Boutet S (2012). High-resolution protein structure determination by serial femtosecond crystallography. Science.

[56] Boutet S, J Williams G (2010). The Coherent X-ray Imaging (CXI) instrument at the Linac Coherent Light Source (LCLS). New J. Phys..

[57] Bunkóczi G (2013). Phaser.MRage: automated molecular replacement. Acta Crystallogr. D. Biol. Crystallogr..

[58] Emsley P, Cowtan K (2004). Coot: model-building tools for molecular graphics. Acta Crystallogr. D. Biol. Crystallogr..

[59] Afonine PV (2012). Towards automated crystallographic structure refinement with phenix.refine. Acta Crystallogr. D. Biol. Crystallogr..

[60] Krissinel E, Henrick K (2004). Secondary-structure matching (SSM), a new tool for fast protein structure alignment in three dimensions. Acta Crystallogr. D. Biol. Crystallogr..

