# Supplementary material for: In cellulo crystallization of Trypanosoma brucei IMP dehydrogenase enables the identification of genuine co-factors
Source: Nat Commun. 2020 Jan 30;11:620. doi: 10.1038/s41467-020-14484-w (PMC6992785; doi:10.1038/s41467-020-14484-w)
Supplement: Supplementary file 1 — Supplementary Information [file 41467_2020_14484_MOESM1_ESM.pdf]

Supplementary Information

In cellulo crystallization of Trypanosoma brucei IMP dehydrogenase enables the identification of genuine co-factors

K. Nass, L. Redecke et al.

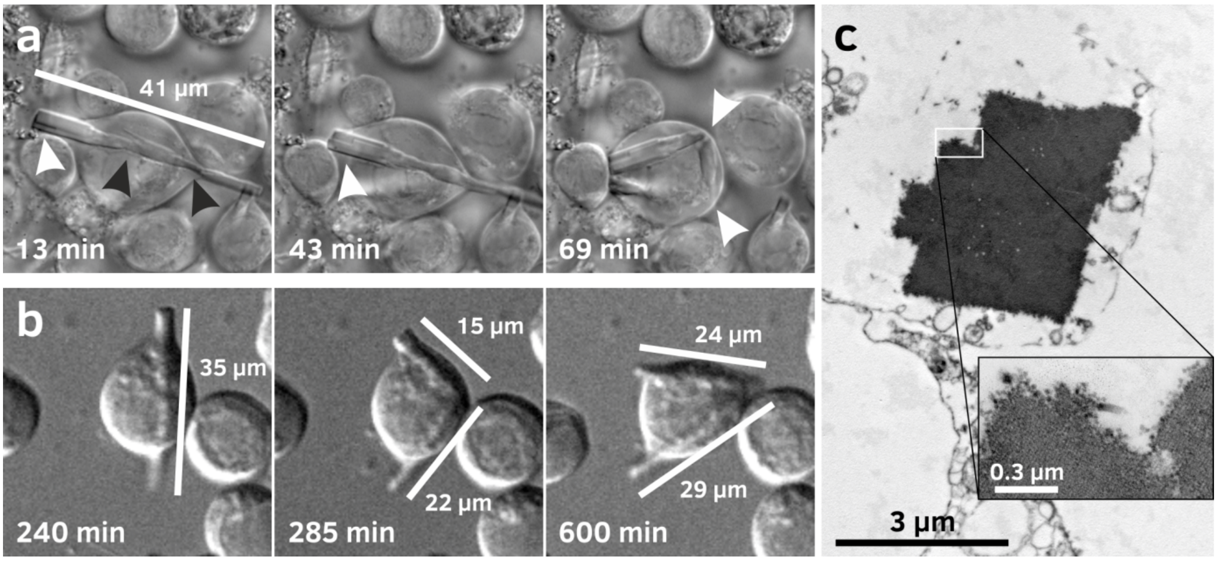

Supplementary Figure 1 Dynamics of TbIMPDH crystals. TbIMPDH crystals show various dynamics within the insect cells. **a)** Crystals can rotate around their long axis (white arrowhead in left and middle panel), as well as break (middle to right panel) at a region of apparent degradation (black arrowheads in left panel). See also **Supplementary Movie 3**. Visible is a Sf9 cell infected with recombinant baculovirus 6 days post infection (p. i.) **b)** After breakage of a crystal, the generated parts can continue to grow. See also **Supplementary Movie 2**. Start of the imaging is 4 days p. i. **c)** Transmission electron micrograph of a TbIMPDH crystal in the remnant of a lysed cell 7 days p. i. showing fuzzy edges, indicating to some extent the beginning of degradation.

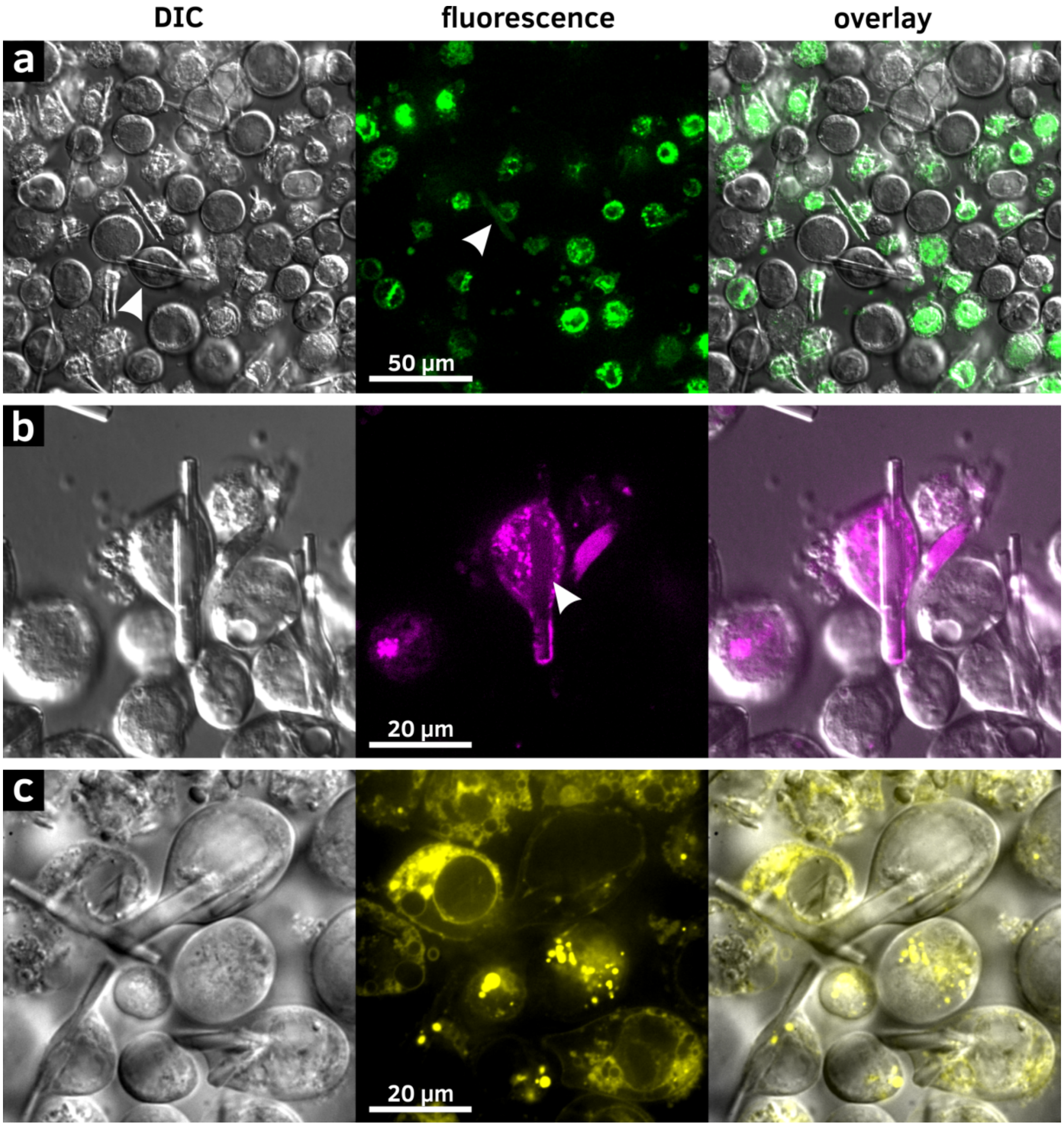
DIC
fluorescence
overlay
a
50 µm
b
20 µm
c
20 µm

Supplementary Figure 2 | Intra- and extracellular localization of TbIMPDH crystals. a) Live-dead-staining of Sf9 cells infected with recombinant baculovirus 8 days p. i. using propidium iodide. Crystals are visible in living (white arrowhead in left panel) and dead cells (white arrowhead in middle panel). In dead cells, a light propidium iodide staining of crystals can be observed (white arrowhead in middle panel). **b)** Co-infection of Sf9 insect cells with recombinant baculoviruses encoding the genes for TbIMPDH and for a Pex26-mCherry fusion protein (marker for peroxisomal membrane) 7 days p. i. Only a negative stain of the crystal is visible within the cell volume (white arrowhead), no enrichment of the marker around the crystal can be observed. **c)** Staining of lysosomes using LysoTracker DeepRed in Sf9 cells infected with recombinant TbIMPDH-encoding baculovirus 7 days. p. i. No staining of crystals with LysoTracker can be observed. Monochrome images of fluorescence channels have been artificially colored for better visibility.

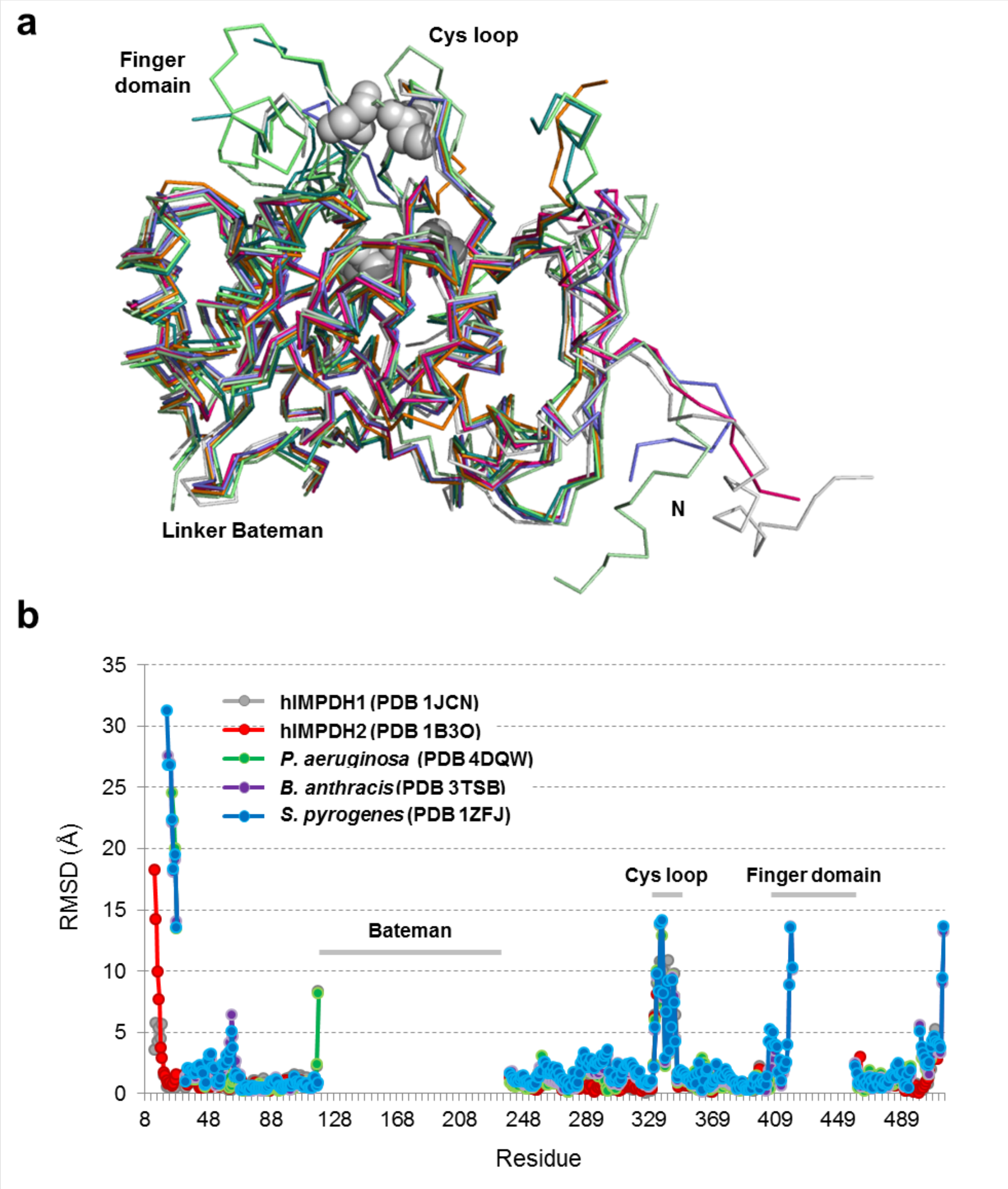

Supplementary Figure 3 | Structural homology of selected IMPDH catalytic domains. a) Ribbon representation of the Cα carbons of the TbIMPDH catalytic domain (*light green*) superimposed to that of human IMPDH1 (*blue*) and IMPDH2 (*red*) as well as on that of P. aeruginosa (*orange*), B. anthracis (*green*), S. pyrogenes (*dark green*), and A. gossypii (*grey*) IMPDH. The catalytically active residues Ser333, Cys336, Asp364, and Gly371 of TbIMPDH are highlighted as grey spheres. **b)** Average RMSD between the residues of the TbIMPDH catalytic domain and of equivalent Cα atoms in the IMPDH structures mentioned in a). Significant structural differences (> 4 Å) are due to the flexible Cys loop, the finger domain, and the N- and C-terminal fragments.

Supplementary Figure 4

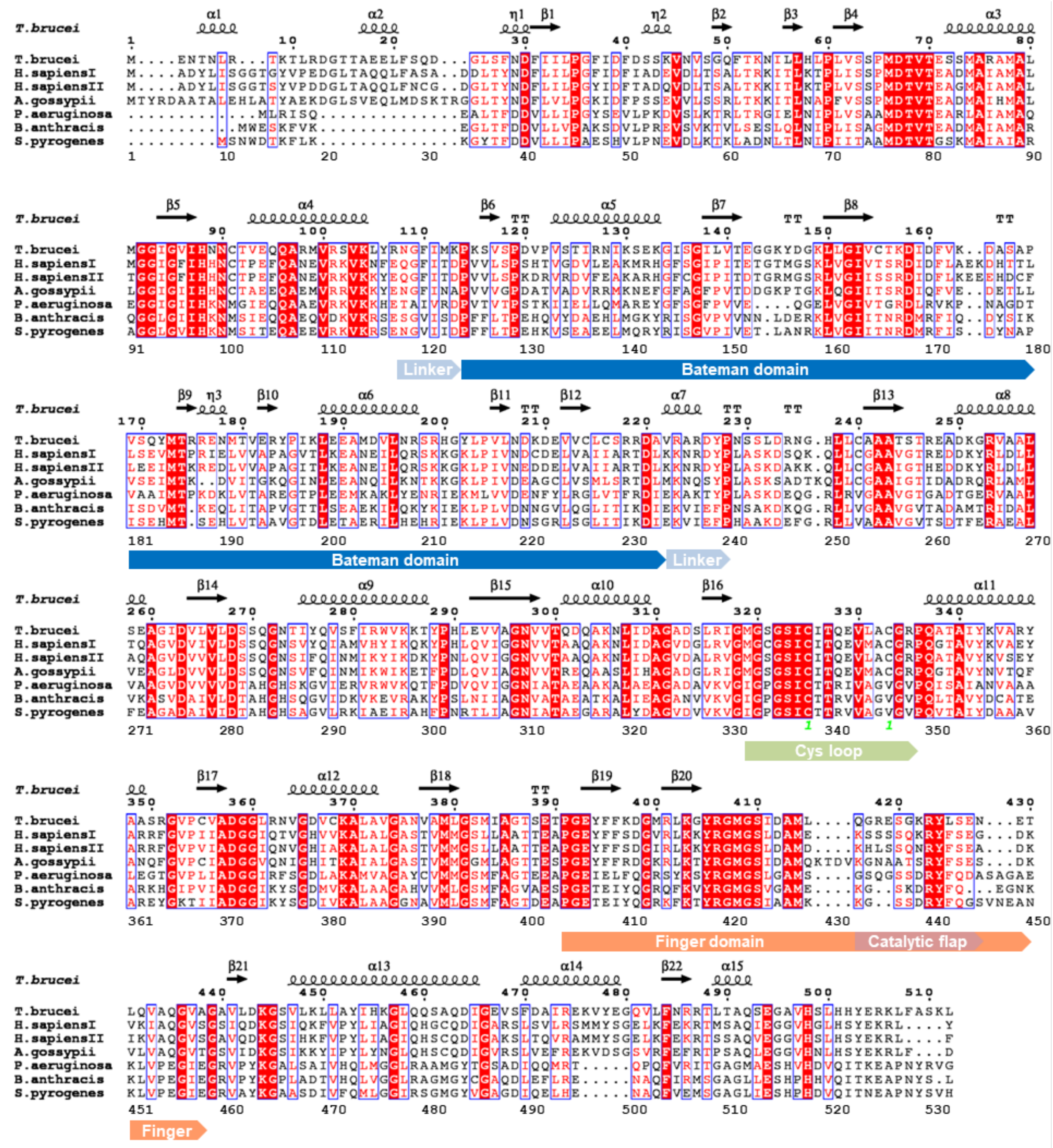

Supplementary Figure 4 | Multiple sequence alignment of IMPDH from selected organisms. Identical amino acids at a specific position are highlighted in red. The individual domains and linker regions are indicated below the sequence alignment, while secondary structure motifs identified in the TbIMPDH structure are depicted above. The upper numbering corresponds to the IMDPH sequence of T. brucei, the lower numbering represents a consensus numbering. The figure was created using ESPript 3[1].

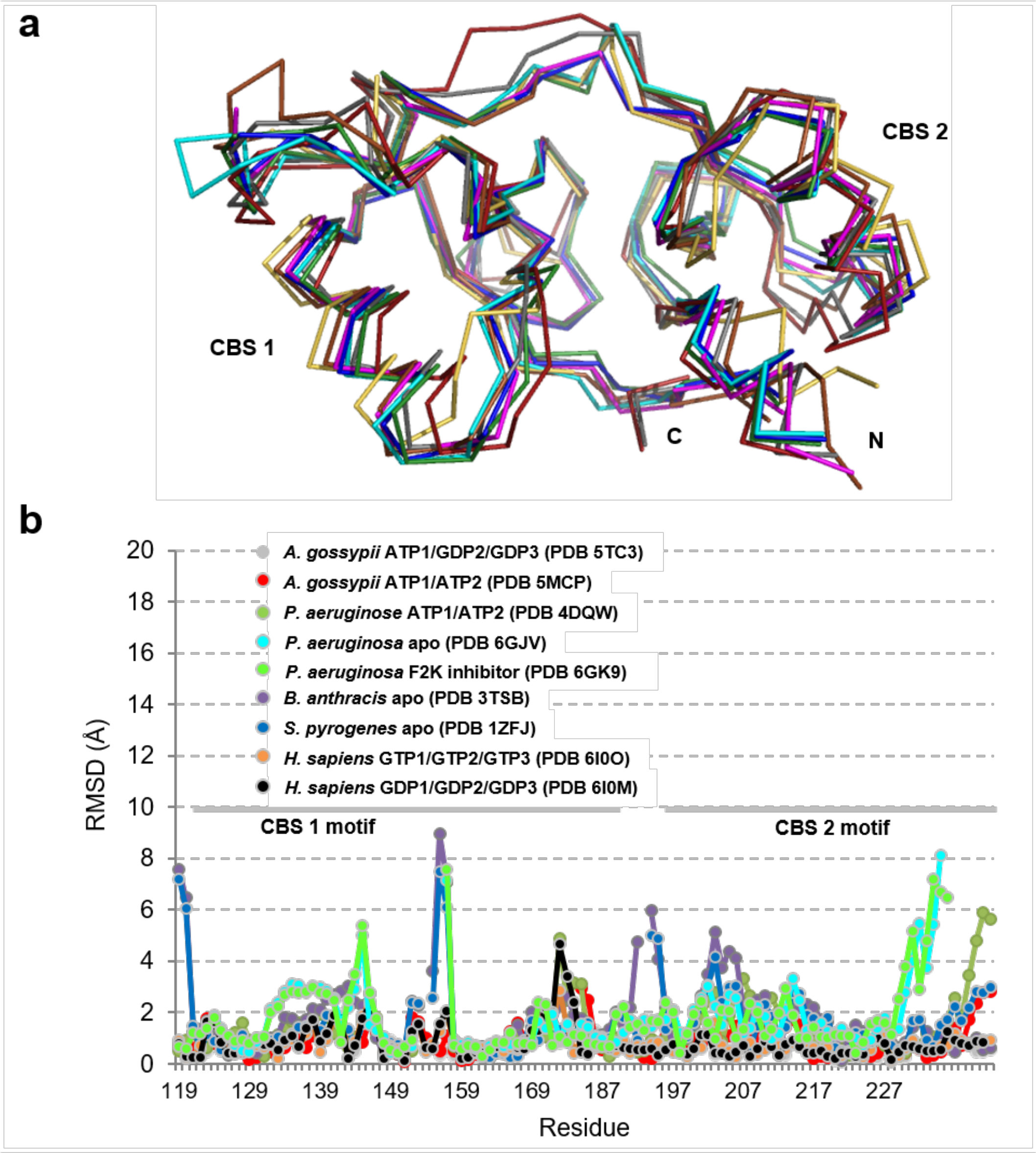

Supplementary Figure 5| Structural homology of IMPDH Bateman domains. a) Ribbon representation of the Cα carbons of the TbIMPDH-ATP1/GMP2 Bateman domain (*green*) superimposed to that of prokaryotic B. antracis (*red*), S. pyrogenes (*grey*), and P. aeruginosa (apo, *yellow*; ATP1/ATP2, *brown*) IMPDH as well as of eukaryotic *A. gossypii* IMPDH (ATP1/GDP2/GDP3, *blue*; ATP1/ATP2 *magenta*) and human IMPDH2-GTP1/GTP2/GTP3 (*cyan*). **b)** Average RMSD between the residues of the TbIMPDH Bateman domain and of equivalent Cα atoms in selected IMPDH structures. Significant structural differences (> 4 Å) are due to the loop linking both CBS motifs and to the N- and C-terminal residues that are located close to the flexible linker sequences that connect the Bateman domains with the catalytic domains.

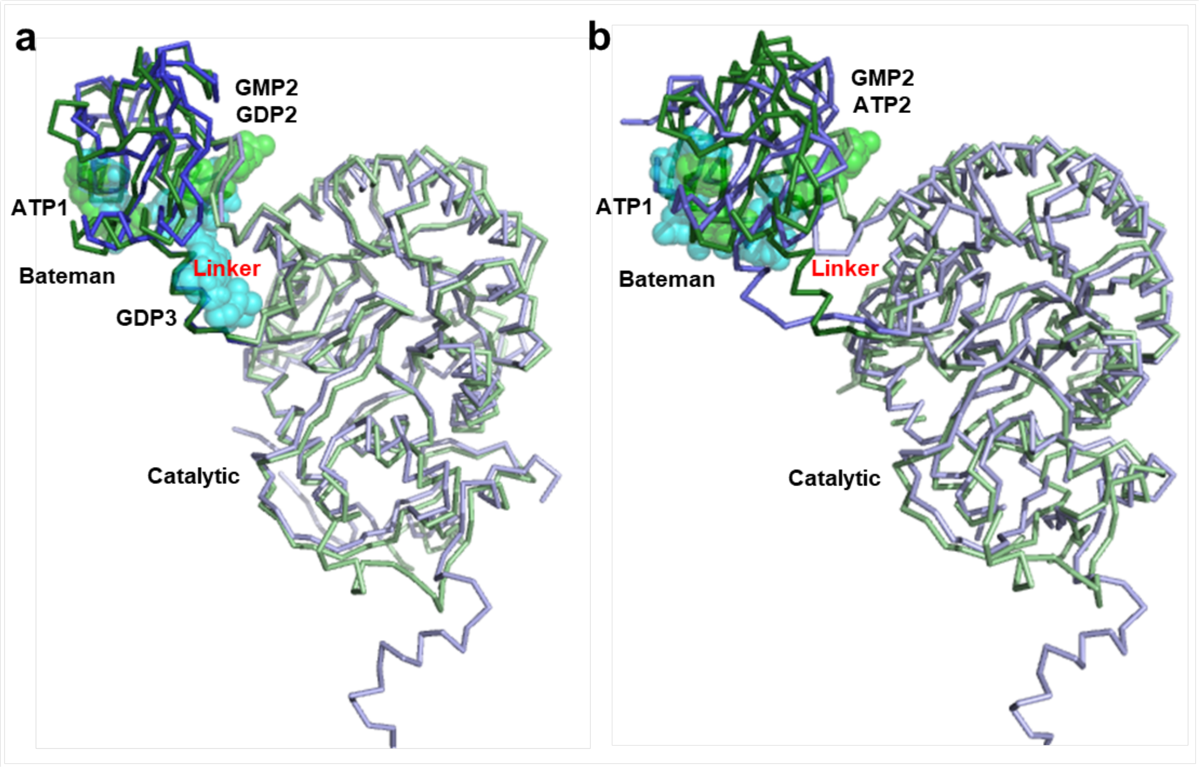

Supplementary Figure 6 | Superposition of guanine nucleotide-bound TbIMPDH and AgIMPDH structures. a) Ribbon representation of the Cα carbons of TbIMPDH-ATP1/GMP2 (*green*) superimposed to that of AgIMPDH-ATP1/GDP2/GDP3 (PDB 5TC3, *blue*). The specific orientation of the regulatory Bateman and the catalytic domain is superimposable in both structures, even the linker regions between the domains share an almost identical conformation. hIMPDH2-GTP1/GTP2/GTP3 (PDB 6I0O) and hIMPDH2-GDP1/GDP2/GDP3 (PDB 6I0M) adopt a comparable domain orientation, as revealed by superposition with the TbIMPDH-ATP1/GMP2 complex (*not shown*). **b)** In contrast, the relative orientation of the Bateman and catalytic domains in ATP-bound AgIMPDH (PDB 5MCP, *blue*) is significantly different, as revealed by the superposed Cα plots of TbIMPDH-ATP1/GMP2 (*green*) and AgIMPDH-ATP1/ATP2 (PDB 5MCP, *blue*). Significant deviations in the linker regions result in a shift of the Bateman domains. Nucleotide atoms are shown as green and blue spheres, respectively.

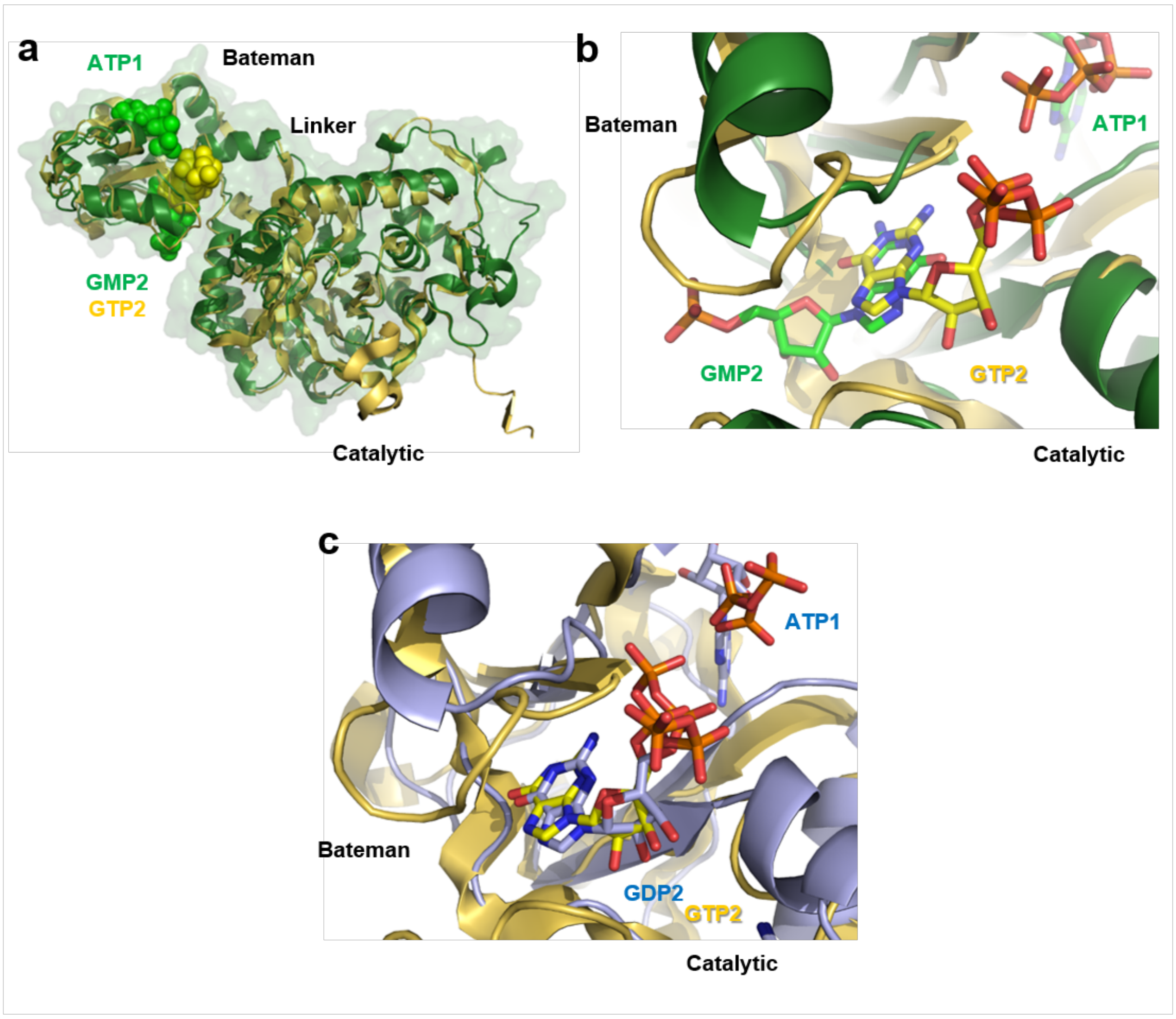

Supplementary Figure 7 | Structural comparison of IMPDH and GMPR structures. a) Cartoon and surface representation of superposed monomers A from TbIMPDH-ATP1/GMP2 (*green*) and from TbGMPR-GTP2 (*yellow,* PDB 5X8O). Nucleotide atoms are shown as green and yellow spheres, respectively. Both structures adopt an almost superimposable relative orientation of the catalytic and the Bateman domain. **b)** Detailed view of the second canonical nucleotide binding site in the Bateman domain, where GMP is bound in TbIMPDH, while TbGMPR shows GTP binding in a flipped conformation. The guanine base is located in the same cavity, but the ribose and phosphate moieties extend into opposite directions. **c)** Superposition of the Bateman domains of AgIMPDH-ATP1/GDP2/GDP3 and TbGMPR-GTP2 reveals an almost identical coordination of GDP and GTP at the second canonical nucleotide binding site. Nucleotide atoms are shown in stick representation in b) and c).

Supplementary Figure 8

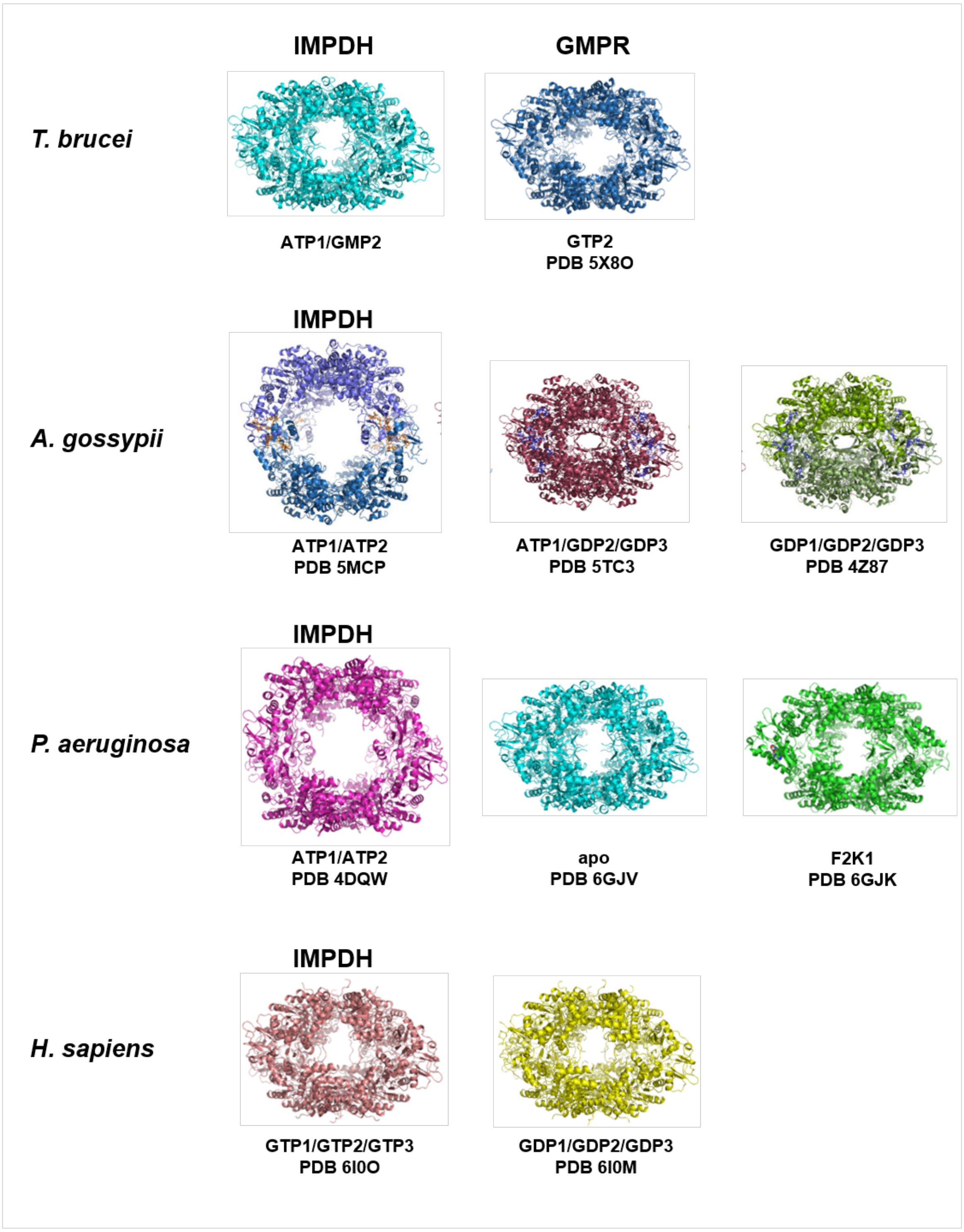

Supplementary Figure 8 | Overview of oligomeric IMPDH and GMPR states. Oligomers formed by IMPDHs from different species in the presence and absence of nucleotides at the canonical binding sites 1 and 2 or at the non-canonical site 3 as well as in the presence of the allosteric inhibitor F2K at site 1 (P. aeruginosa) are presented in cartoon representation. The oligomer structure of TbGMPR-GTP2 is shown for comparison. Extended oligomer conformations are only observed if ATP binds to the second canonical nucleotide binding site.

Supplementary Table 1

Electron and photon beam parameters for the SFX experiment of TbIMPDH in cellulo crystals at the CXI instrument (LCLS)

Parameter	Mean
Electron energy* [MeV]	14.6570
Charge* [nC]	0.1466
Electron pulse duration* [fs]	52.2404
Photon pulse energy* [mJ]	2.4159
Photon energy* [eV]	9510
Photon wavelength* [Å]	1.299
Power density at the sample** [Wcm^{-2}]	$1.9x10^{17}$

* Varies from shot to shot, not including beamline transmission.
** Focus 4 μm^2, 15 % beamline transmission included.

Supplementary Table 2

Data collection statistics in resolution shells for the SFX experiment of TbIMPDH in cellulo crystals at the CXI instrument (LCLS)

Min d (Å)	Max d (Å)	CC 1/2	CC*	R_{split}[a] (%)	SNR	Compl.	Redund.
52.632	6.901	0.963	0.991	11.48	9.06	100	225
6.901	5.479	0.954	0.988	12.95	7.61	100	152
5.479	4.787	0.960	0.990	13.10	7.49	100	119
4.787	4.350	0.964	0.991	13.21	7.30	100	107
4.350	4.037	0.959	0.989	14.56	6.54	100	100
4.037	3.799	0.956	0.989	15.97	5.90	100	97
3.799	3.610	0.919	0.979	21.40	4.73	100	90
3.610	3.453	0.871	0.965	28.56	3.68	100	81
3.453	3.320	0.826	0.951	33.54	3.14	100	79
3.320	3.205	0.698	0.907	44.71	2.53	100	79
3.205	3.105	0.581	0.857	53.86	2.14	100	80
3.105	3.017	0.517	0.826	66.11	1.77	100	74
3.017	2.937	0.393	0.751	81.05	1.43	100	69
2.937	2.865	0.282	0.663	87.63	1.33	100	61
2.865	2.800	0.199	0.576	114.35	1.00	100	52

[a] $R_{split} = 2^{-1/2} \frac{\sum|I_{even} - I_{odd}|}{\frac{1}{2}\sum(I_{even} - I_{odd})}$

Supplementary Table 3

Comparison of full-length TbIMPDH as well as of the individual catalytic and Bateman domains in terms of sequence and the structure with that of selected pro- and eukaryotic IMPDH as well as of T. brucei GMP reductase (GMPR). Protein ID, UniProtKB accession number; PDB#, Protein Data Base accession number; AA, number of amino acids; No. identical, number of amino acids identical to TbIMPDH; % identical, percentage of amino acids identical to TbIMPDH; RMSD, root-mean-square deviation of equivalent Cα residues determined by superposition with the TbIMPDH structure. #In contrast to all other structures, the B domain of 1B3O was used for structural comparison due to the significantly increased completeness compared to the associated A domain.

IMPDH	Organism	Protein ID	PDB#	AA	No. identical	% identical	RMSD [Å]
**Full length**	T. brucei	P50098		512	-	-	-
	T. brucei GMPR	Q57ZS7	5X8O	491	169	34.4	1.98 ± 2.71
	H. sapiens 1	P20839	1JCN	514	272	52.5	3.04 ± 2.07
	H. sapiens 2	P12268	1B3O#	514	268	51.7	6.06 ± 5.21
	A. gossypii	Q756Z6	4XWU	522	271	51.4	1.10 ± 2.01
	P. aeruginosa	Q9HXM5	4DQW	489	184	37.7	3.68 ± 3.97
	B. anthracis	A0A0J1HJU0	3TSB	487	179	36.7	2.61 ± 3.57
	S. pyrogenes	M6KEW3	1ZFJ	493	179	35.3	3.36 ± 4.12
**Catalytic domain**	T. brucei	P50098		403	-	-	-
	T. brucei GMPR	Q57ZS7	5X8O	380	141	37.1	1.31 ± 0.80
	H. sapiens 1	P20839	1JCN	404	228	56.4	0.81 ± 1.07
	H. sapiens 2	P12268	1B3O#	404	231	57.2	1.78 ± 1.82
	A. gossypii	Q756Z6	4XWU	415	230	55.4	1.10 ± 2.01
	P. aeruginosa	Q9HXM5	4DQW	384	155	40.4	1.91 ± 4.22
	B. anthracis	A0A0J1HJU0	3TSB	380	152	40.0	1.92 ± 4.00
	S. pyrogenes	M6KEW3	1ZFJ	386	149	38.6	2.30 ± 4.63
**Bateman domain**	T. brucei ATP1/GMP2	P50098		109	-	-	-
	T. brucei GMPR GTP2	Q57ZS7	5X8O	111	28	25.2	2.19 ± 1.88
	H. sapiens 1 apo	P20839	1JCN	110	44	40.0	n.d.
	H. sapiens 2						
	GTP1/GTP2/GTP3	P12268	6I0O	110	37	33.6	0.81 ± 0.49
	GDP1/GDP2/GDP3	P12268	6I0M	110	37	33.6	0.84 ± 0.65
	A. gossypii						
	ATP1/ATP2	Q756Z6	5MCP	107	41	38.3	0.93 ± 0.59
	ATP1/GDP2/GDP3	Q756Z6	5TC3	107	41	38.3	0.70 ± 0.40
	P. aeruginosa						
	apo	Q9HXM5	6GJV	105	29	27.1	1.93 ± 1.79
	F2K inhibitor	Q9HXM5	6GK9	105	29	27.1	1.79 ± 1.42
	ATP1/ATP2	Q9HXM5	4DQW	105	29	27.1	1.43 ± 1.18
	B. anthracis apo	A0A0J1HJU0	3TSB	107	27	25.2	1.83 ± 1.55
	S. pyrogenes apo	M6KEW3	1ZFJ	107	30	28.0	1.63 ± 1.31

Supplementary Table 4

Interfaces and main stabilizing interactions in TbIMPDH-ATP1/GMP2 assemblies. Interactions have been detected and scored by interface analysis using the PDBePISA server (www.ebi.ac.uk/pdbe/pisa/). BSA, buried surface area; H, hydrogen bond; S, salt bridge.

	Monomer A	Distance	Monomer B	Interaction
Dimer in ASU<br>Interface 1<br>AB[ATP]$_2$[GMP]$_2$<br>BSA = 5.500 Å²<br>ΔG_{diss} = -0.5 kcal/mol	ASP 160 [OD1]	2.40	ARG 218 [HH12]	H
	ASP 160 [OD2]	2.06	ARG 218 [HH11]	H
	ARG 199 [HE]	2.31	ASP 158 [OD1]	H
	ARG 218 [HH12]	2.13	ASP 160 [OD1]	H
	ARG 218 [HH11]	2.20	ASP 160 [OD2]	H
	ARG 199 [HH21]	2.16	TYR 172 [O]	H
	ASP 158 [OD1]	3.32	ARG 199 [NH1]	S
	ASP 160 [OD1]	2.78	ARG 218 [NH1]	S
	ASP 160 [OD2]	2.76	ARG 218 [NH1]	S
	ARG 199 [NE]	3.09	ASP 158 [OD1]	S
	ARG 218 [NH1]	2.66	ASP 160 [OD1]	S
	ARG 218 [NH1]	2.76	ASP 160 [OD2]	S
Tetramer A<br>Interface 1<br>A$_4$[ATP]$_4$[GMP]$_4$<br>BSA = 14.680 Å²<br>ΔG_{diss} = 7.6 kcal/mol	Monomer A$_1$	Distance	Monomer A$_2$	Interaction
	ASN 306 [HD21]	2.26	LEU 6 [O]	H
	ARG 335 [HH22]	2.29	ASP 13 [OD1]	H
	ARG 335 [HH12]	1.97	ASP 13 [OD2]	H
	GLN 301 [H]	2.26	ASP 13 [OD2]	H
	GLN 271 [HE21]	2.29	GLY 36 [O]	H
	GLN 271 [HE22]	2.14	GLY 36 [O]	H
	ASN 273 [HD22]	2.04	GLY 36 [O]	H
	ILE 275 [H]	2.10	ILE 38 [O]	H
	GLY 25 [H]	1.93	ARG 486 [O]	H
	ARG 362 [NH2]	2.40	GLU 494 [OE2]	H
	ASP 30 [OD2]	2.46	THR 488 [OG1]	H
	ARG 335 [NH1]	3.85	ASP 13 [OD1]	S
	ARG 335 [NH2]	3.14	ASP 13 [OD1]	S
	ARG 335 [NH1]	2.80	ASP 13 [OD2]	S
	ARG 335 [NH2]	3.59	ASP 13 [OD2]	S
	ARG 362 [NH2]	3.58	GLU 494 [O]	S

	Monomer B_1	Distance	Monomer B_2	Interaction
Tetramer B<br><br>Interface 1<br><br>$B_4[ATP]_4[GMP]_4$<br>BSA = 15.170 Å²<br>ΔG_{diss} = 6.4 kcal/mol	ARG 362 [NH2]	2.40	GLU 494 [OE2]	S
	ARG 12[H]	2.04	ASP 302[OD1]	H
	ASP 13[H]	1.95	ASP 302[OD2]	H
	ILE 38[H]	2.17	ASN 273[O]	H
	THR 488[H]	2.28	GLY 25[O]	H
	THR 488[OG1]	2.50	ASP 30[OD2]	H
	ASP 13[OD1]	2.94	ARG 335[NH2]	S
	ASP 13[OD1]	3.69	ARG 335[NH1]	S
	ASP 13[OD2]	3.51	ARG 335[NH2]	S
	ASP 13[OD2]	2.70	ARG 335[NH1]	S

Supplementary Table 5

Comparison of dimeric, tetrameric, and octameric assemblies formed by IMPDH of different species. Interfaces have been analyzed using the PDBePISA server (www.ebi.ac.uk/pdbe/pisa/).

Organism<br>(PDB code)	ASU	Dimer<br>composition<br>surface area [Å²]<br>buried area [Å²]<br>ΔG^{diss} [kcal/mol]<br>stability[1]	Tetramer 1<br>composition<br>surface area [Å²]<br>buried area [Å²]<br>ΔG^{diss} [kcal/mol]<br>stability[1]	Tetramer 2<br>composition<br>surface area [Å²]<br>buried area [Å²]<br>ΔG^{diss} [kcal/mol]<br>stability[1]	Octamer<br>composition<br>surface area [Å²]<br>buried area [Å²]<br>conformation<br>ΔG^{diss} [kcal/mol]<br>stability[1]
T. brucei<br>(this study)	dimer	$AB[ATP]_2[GMP]_2$<br>38.580<br>5.500<br>-0.5<br>no	$A_4[ATP]_4[GMP]_4$<br>72.910<br>14.680<br>7.6<br>yes	$B_4[ATP]_4[5GP]_4$<br>73.570<br>15.170<br>6.4<br>yes	$A_4B_4[ATP]_8[5GP]_8$<br>136.030<br>40.300<br>compact<br>52.7<br>yes
A. gossypii<br>(5MCP)<br>Buey et al.,<br>2017	octamer	none	$ABCD[ATP]_{12}$<br>70.540<br>23.760<br>71.2<br>yes	$EFGH[ATP]_{12}$<br>69970<br>16640<br>39.3<br>yes	$ABCDEFGH[ATP]_{24}$<br>136.920<br>44.020<br>extended<br>-1.3<br>maybe
A. gossypii<br>(4Z87)<br>Buey et al.,<br>2015	tetramer	$AB[GMP]_2[GDP]_6$<br>44.540<br>8.640<br>10.3<br>yes	$A_4[GMP]_4[GDP]_8$<br>77.030<br>27.980<br>286.9<br>yes	$B_4[GMP]_4[GDP]_{16}$<br>75.350<br>32.340<br>291.0<br>yes	$A_4B_4[GMP]_8[GDP]_{24}$<br>136.160<br>76.540<br>compact<br>81.8<br>yes
A. gossypii<br>(5TC3)<br>Buey et al.,<br>2017	dimer	-	$A_4[ATP]_4[GDP]_8$<br>72.560<br>31.190<br>92.0<br>yes	$B_4[ATP]_4[GDP]_8$<br>72.430<br>29.880<br>79.2<br>yes	$A_4B_4[ATP]_8$[GDP]16<br>PISA: not stable<br>compact
B. antracis<br>(3USB)<br>Makowska-<br>Grzyska et<br>al., 2012	dimer	$AB[IMP]_2$<br>-<br>1.014<br>-6.7<br>no	$A_4[IMP]_4$<br>72.860<br>18.760<br>68.2<br>yes	$B_4[IMP]_4$<br>70.910<br>19.850<br>98.2<br>yes	$A_4B_4[IMP]_8$<br>135.650<br>46.730<br>22.6<br>yes
B. antracis<br>(3TSD)<br>Makowska-<br>Grzyska et<br>al., 2012	dimer	$AB[XMP]_2$<br>-<br>898<br>-3.5<br>no	$A_4[XMP]_4$<br>71.380<br>17.260<br>73.3<br>yes	$B_4[XMP]_4$<br>77.550<br>19.350<br>74.9<br>yes	$A_4B_4[XMP]_8$<br>141.650<br>43.890<br>16.2<br>yes
P.<br>aeruginosa<br>(4DQW)<br>Labesse et<br>al., 2013	dimer	$AB[ATP]_4$<br>-<br>561<br>1.9<br>maybe	$A_4[ATP]_8$<br>71.390<br>20.220<br>65.5<br>yes	$B_4[ATP]_8$<br>66.660<br>17.810<br>54.9<br>yes	$A_4B_4[ATP]_{16}$<br>130.680<br>45.400<br>extended<br>81.7<br>yes
P.<br>aeruginosa<br>(6GJV)<br>Alexandre<br>et al., 2019	octamer	AC/FD<br>38.890/32.700<br>2.820/2.830<br>15.6/15.1<br>yes	BEGH<br>66.480<br>11.070<br>42.1<br>yes	ACFD<br>65.97ß<br>11.270<br>40.8<br>yes	$A_4B_4[ATP]_{16}$<br>123.610<br>31.180<br>compact<br>50.9<br>yes

P. aeruginosa (6GK9) Alexandre et al., 2019	octamer	-	ACDF[F2K]2<br>65.850<br>12.360<br>62.8<br>yes	BEGH<br>68.260<br>11.290<br>64.7<br>yes	$ABCDEFGH[F2K]_2$<br>124.540<br>33.220<br>compact<br>167.9<br>yes
human (6I0O) Fernandez-Justel et al., 2019	dimer	$AB[GTP]_6$<br>37.060<br>11.550<br>4.6<br>yes	$A_4[GTP]_{12}$<br>66.830<br>27.570<br>50.3<br>yes	$B_4[GTP]_{12}$<br>68.450<br>31.600<br>75.7<br>yes	$A_4B_4[GTP]_{24}$<br>126.460<br>67.990<br>compact<br>58.3<br>yes
human (6I0M) Fernandez-Justel et al., 2019	dimer	$AB[GMP]_2$[GDP]6<br>38.560<br>10.290<br>8.9<br>yes	$A_4[GMP]_4[GDP]_{12}$<br>71.150<br>28.480<br>66.1<br>yes	$B_4[GMP]_4[GDP]_{12}$<br>71.950<br>23.840<br>35.6<br>yes	$A_4B_4[GMP]_8[GDP]_{24}$<br>130.460<br>64.970<br>compact<br>85.4<br>yes
T. brucei GMPR (5X0O) not published	monomer	none	$A_4[GTP]_4$<br>66.710<br>14.680<br>53.0<br>yes	none	$A_8[GTP]_8$<br>128.000<br>34.780<br>compact<br>19.1<br>yes

[1]Stability estimation based on calculated free energy of assembly dissociation ΔG^{diss} [kcal/M]. Assemblies with $\Delta G^{diss}>0$ are estimated to be thermodynamically stable.

Supplementary References

1. Robert, X. & Gouet, P. Deciphering key features in protein structures with the new ENDscript server. *Nucleic Acids Res*. **42**, W320-4 (2014).
