# Supplementary material for: In cellulo crystallization of Trypanosoma brucei IMP dehydrogenase enables the identification of genuine co-factors
Source: Nat Commun. 2020 Jan 30;11:620. doi: 10.1038/s41467-020-14484-w (PMC6992785; doi:10.1038/s41467-020-14484-w)
Supplement: Supplementary file 2 — Description of Additional Supplementary Files [file 41467_2020_14484_MOESM2_ESM.pdf]

Description of Additional Supplementary Files

File Name: Supplementary Movie 1
Description: Sf9 cells infected with a recombinant baculovirus were imaged starting 5 days after infection. Crystal growth is simultaneously observed in two dimensions over the time course of several hours.

File Name: Supplementary Movie 2
Description: Sf9 cells infected with a recombinant baculovirus were imaged starting 4 days after infection. Crystal growth continues for several hours within the depicted Sf9 cell. As a result of the increasing tension on the plasma membrane the crystal breaks and the generated parts continue to grow without disturbance.

File Name: Supplementary Movie 3
Description: Imaging of baculovirus infected Sf9 cells was performed 6 days after initial infection. Crystals can slowly rotate around their long axis within the living cell as well as break at regions of apparent degradation.

File Name: Supplementary Movie 4
Description: Sf9 insect cells were co-infected with recombinant baculoviruses containing the genes for TbIMPDH and peroxisomal EGFP-SKL. Imaging was performed 8 days after co-infection. EGFP-SKL shows cytoplasmatic fluorescence as well as local enrichment, identified as peroxisomes. Plasma membrane disruption is induced by addition of hypotonic buffer at the 0 s-timepoint. EGFP fluorescence is quickly lost from the cytoplasm and the crystal volume, whereas peroxisomes remain undisturbed.
